# The Interplay of Strongly and Weakly Exchange-Coupled Triplet Pairs in Intramolecular Singlet Fission

**DOI:** 10.1021/jacs.4c10483

**Published:** 2024-10-17

**Authors:** Oliver Millington, Stephanie Montanaro, Ashish Sharma, Simon A. Dowland, Jurjen Winkel, Jeannine Grüne, Anastasia Leventis, Troy Bennett, Jordan Shaikh, Neil Greenham, Akshay Rao, Hugo Bronstein

**Affiliations:** † Department of Chemistry, University of Cambridge, Cambridge, CB2 1EW, U.K.; ‡ Cavendish Laboratory, University of Cambridge, Cambridge, CB3 0HE, U.K.

## Abstract

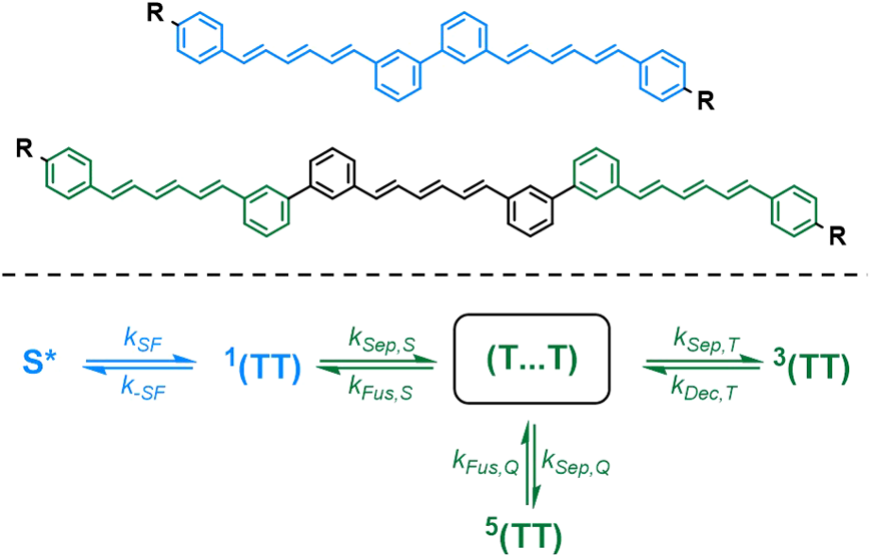

Singlet fission (SF) and triplet–triplet annihilation upconversion (TTA-UC) nominally enable the interconversion of higher-energy singlet states with two lower-energy triplet states and vice versa, with both processes having envisaged application for enhanced solar power devices. The mechanism of SF/TTA-UC involves a complex array of different multiexcitonic triplet-pair states that are coupled by the exchange interaction to varying extents. In this work a family of bounded intramolecular SF materials, based upon the chromophore 1,6-diphenyl-1,3,5-hexatriene, were designed and synthesized. Their SF behavior was characterized using fluorescence lifetime, transient absorption, and magnetic field dependence studies. The capacity for the formation of weakly exchange-coupled triplet pairs, and subsequent spin-evolution, is shown to be strongly dependent upon the combined factors of oligomer size and geometry. By contextualizing these results with the wider SF literature, we present a general schematic model for SF/TTA-UC of greater completeness than portrayed elsewhere.

## Introduction

The maximum efficiency of a single-junction photovoltaic device for solar power generation is fundamentally constrained by the Shockley-Queisser (SQ) limit.^1,2^

Singlet fission (SF) and the reverse process of triplet–triplet annihilation upconversion (TTA-UC) each present a potential mechanism for surpassing the SQ-limit of photovoltaic (PV) power devices.^3^ By converting one high energy singlet state to two lower energy triplet states in an SF-active material, and harvesting both separately, key energetic losses arising from the thermalization of high-energy photons in the PV semiconductor material could be reduced.^4−6^ Meanwhile, losses arising from the inability of a semiconductor to absorb photons below its bandgap may be reduced via the conversion of two low-energy photons to a single higher energy photon in a TTA-UC system.^7−10^ Beyond PV, TTA-UC materials may be leveraged to make use of nonemissive triplet states in organic light emitting diodes (OLEDs),^11−13^ while SF may find application in quantum information.^14−16^

However, the SF and TTA-UC processes involve a complex array of multiexcitonic triplet-pair states. An incomplete understanding of the underlying mechanisms is one of the fundamental challenges that frustrates the rational design of better SF and TTA-UC materials, toward realizing effective application in devices. Advancing understanding of the various excitation decay processes and interconversion pathways between the multiexcitonic states involved in the SF/TTA-UC mechanisms is thus critical to future progress in this field.

In this regard it is necessary to appreciate the distinction between correlated triplet pairs that are strongly coupled by the exchange interaction, parametrized by the exchange energy (*J*_*Ex*_), and triplet pairs that are only weakly exchange coupled.^17−21^ The former are inherently “pure-spin” states i.e. are states for which the total spin can be described by a single spin quantum number, *S*; they are denoted ^M^(TT) where M indicates the spin multiplicity (M = 2*S* + 1). Spin conservation necessitates that the initial product of SF, or conversely the final intermediate for TTA-UC, is a strongly exchange-coupled triplet-pair that has singlet spin multiplicity.^19,22^ Thus, the interconversion of this state, denoted ^1^(TT), with the wider manifold of triplet-pair states of various spin nature is of vital importance to the SF or TTA-UC processes.

Beyond ^1^(TT), there are eight further solutions to the two-triplet spin Hamiltonian in the case of strong exchange-coupling.^20,21,23,24^ These comprise of three states with *S* = 1 and five states with *S* = 2, that can be grouped by spin multiplicity and collectively referred to as ^3^(TT) and ^5^(TT) respectively.

Typical condensed-phase (or solution-state) intermolecular SF and TTA-UC systems can be considered to present an unbounded scenario. While some fraction of triplets generated by SF may undergo geminate recombination,^27,28^ triplet exciton diffusion in the condensed-phase (or molecular diffusion in solution) can facilitate separation to the noninteracting limit (*J*_*ex*_ → 0). This produces the result commonly referred to as “free triplets” and denoted by “T_1_ + T_1_”. The situation is then akin to the starting point in a TTA-UC system, wherein independent free triplets are initially generated. Howsoever they are generated, diffusional encounters of free-triplets may then lead to nongeminate TTA at long time scales. Closely studying the nature of any intermediate triplet-pair states in the nongeminate TTA pathway is complicated by the fact that such states typically have only a fleeting existence relative to their diffusional formation time. Meanwhile in intermolecular SF systems, unpicking the precise significance of interconversions between the strong and weak exchange regimes is complicated by any bleed out of the triplet population to the zero-exchange “free triplet” limit.

Covalently connected dimers and small oligomers of SF active chromophores present inherently bounded systems; the capacity for irreversibly achieving the zero-interaction limit of triplet dissociation is inhibited by the small number and limited physical separation of chromophore sites. Consequently, such materials present an ideal platform with which to study the interconversion of triplet-pair states between the strong and weak exchange regimes and the resulting impact on triplet-pair annihilation pathways. Furthermore, the toolkit of organic synthesis enables tuning of molecular geometry to investigate the influence of chromophore arrangement on these processes.

**Figure 1:**
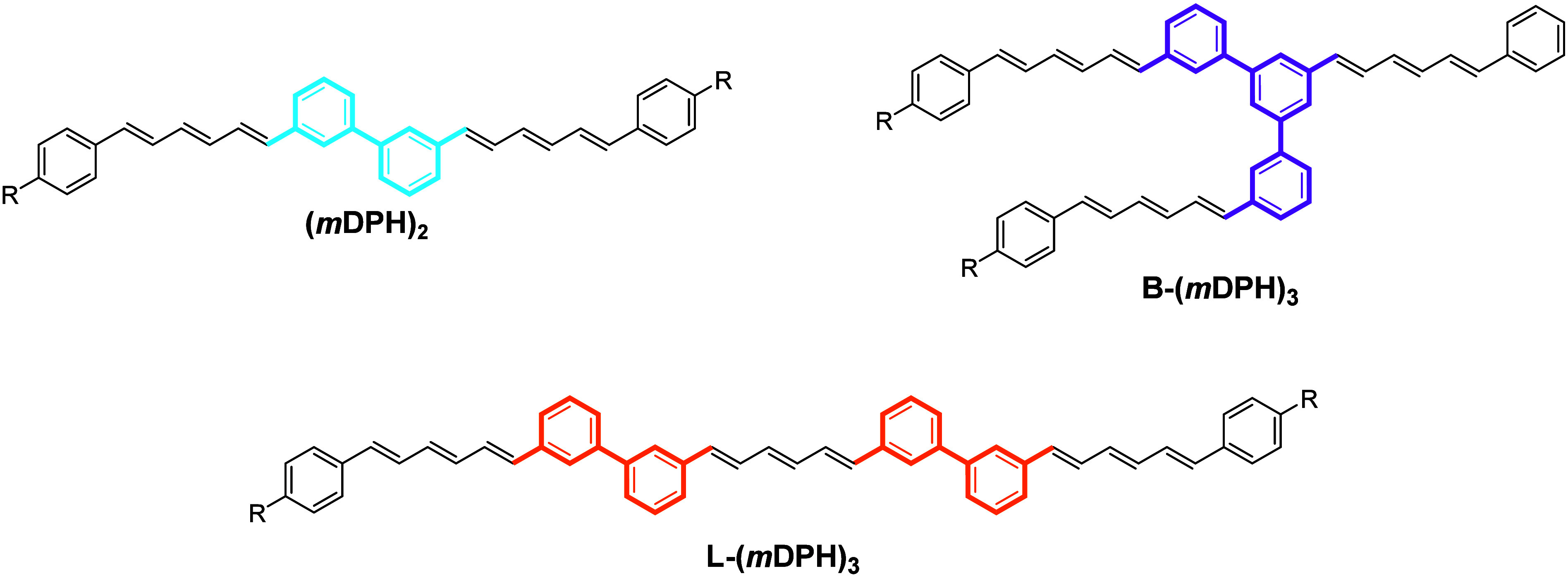
Structure of the contiguous DPH dimer and both branched and linear trimers that are studied in this work. R = 2-ethylhexyl.

Herein, triplet-pair separation and geminate fusion are probed via the experimental study of a novel trio of intramolecular singlet fission (iSF) materials based upon the SF active chromophore diphenylhexatriene (DPH). The set consists of a DPH dimer and two derivative trimers that exhibit branched and linear molecular geometries (Figure 1). The trimers are designed such that they both maintain the connectivity of the dimer between each pair of adjacent DPH chromophores, but the relationship of the terminal chromophores differs. Their iSF behavior is studied through the complementary techniques of transient absorption spectroscopy and fluorescence lifetime studies, with the investigation of magnetic field effects on both. A strong dependence on the overall geometry is observed, with markedly enhanced spin relaxation of the separated triplet-pairs formed in the linear trimer relative to its branched isomer. By considering our experimental results in the context of the body of relevant literature, we present an expanded model of the process of SF with emphasis on the active elements in bounded (i.e., intramolecular) systems.

## Results

### Molecular Design and Synthesis

**Figure 2:**
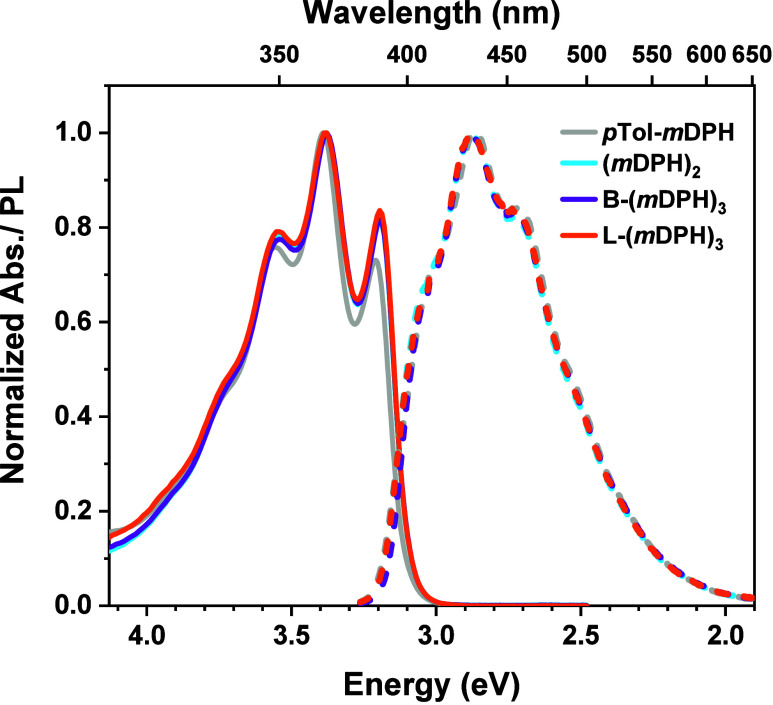
Steady-State Absorption and Photoluminescence (PL) spectra of ultradilute (∼3 μM) samples of the oligomers in toluene in a 1 cm path length fluorescence cuvette. PL spectra were recorded using excitation at 375 nm. Spectra for an exemplar monomeric DPH material, *p*Tol-*m*DPH, are additionally plotted for comparison (data previously published by the authors in ref (29), Copyright © 2023 Millington et al.). The structure of *p*Tol-*m*DPH is analogous to the dimer except that the second DPH chromophore is replaced by a *para*-tolyl group.

Our previous study of phenylene-linked DPH dimers revealed that optimal chromophore arrangements for iSF in DPH assemblies should feature a moderate degree of conformational flexibility and avoid excessively strong conjugative through-bond coupling of the hexatrienes.^29^ The simplest dimer unit that could satisfy these criteria is the contiguously connected dimer (*m*DPH)_2_. In (*m*DPH)_2_ two DPH units are directly connected without a linker but direct conjugative communication between the two hexatriene units is broken by *meta*-type (“*m*”) substitution arrangements on the central phenylene rings. The steady-state absorption and photoluminescence spectra of (*m*DPH)_2_ are highly consistent with the spectra of a monomeric reference DPH derivative (Figure 2). This demonstrates that the interchromophore coupling is not excessive in the dimer, as in the limit of strong coupling the spectral onsets would be anticipated to shift significantly.

Beyond the dimer, there are two possible trimeric structures that maintain the coupling arrangement of the dimer between each pair of DPH units. A branched trimer, B-(*m*DPH)_3_, is achieved by coupling two *m*DPH units to the same end of a third DPH unit so that they are both *meta* to one another and also the hexatriene of the central DPH. Meanwhile, an isomeric linear trimer, L-(*m*DPH)_3_, is produced by coupling three DPH units end to end, with each pair connected in the manner of the dimer. Terminal 2-ethylhexyl groups were incorporated into the final structures in order to aid the solubility of the final materials.

### Photophysical Behavior of the Dimer

The first step of the singlet fission mechanism was investigated using femtosecond transient absorption spectroscopy (fsTA).

**Figure 3:**
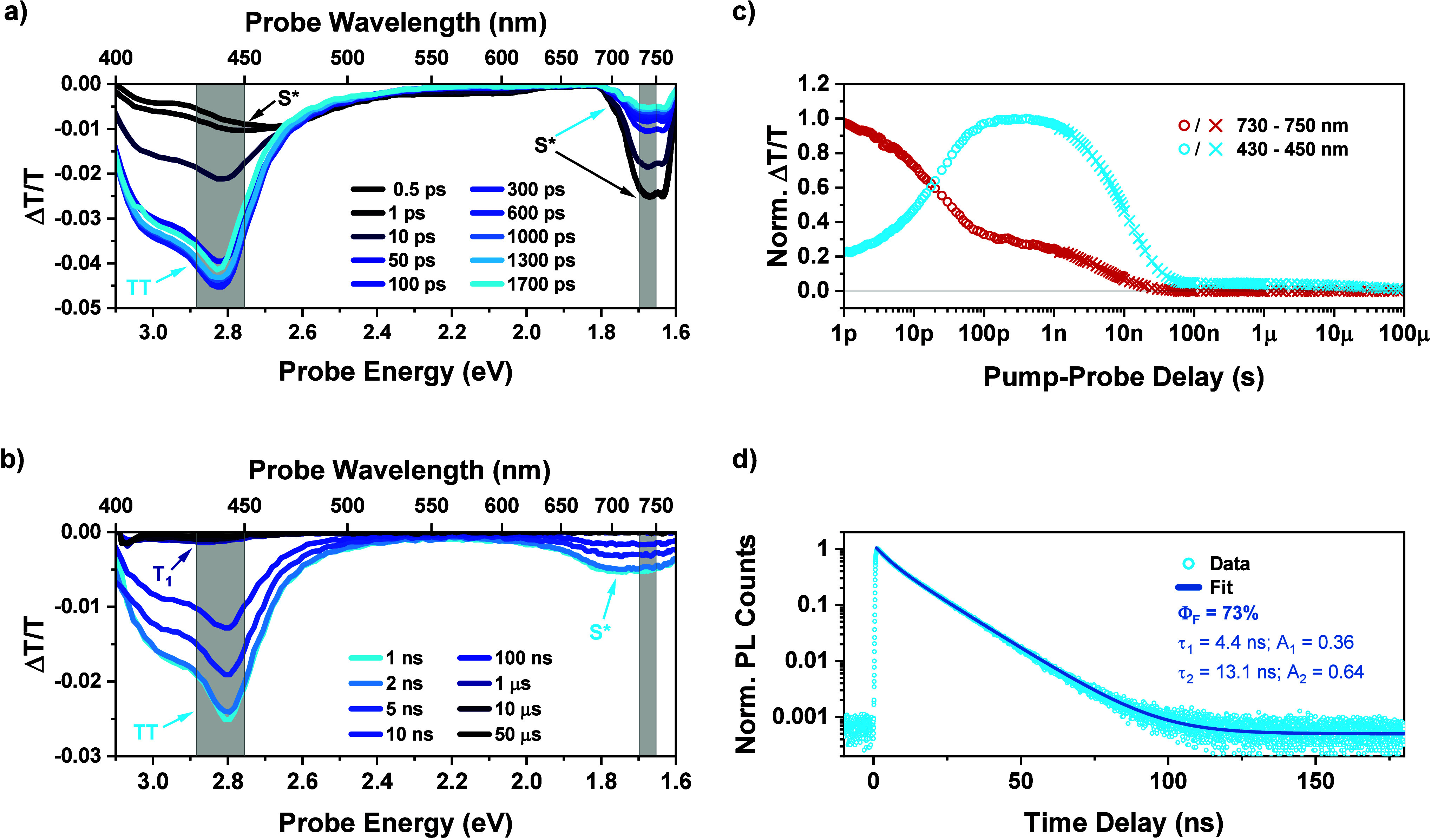
Spectra and Kinetics for 1 mM Solution of (*m*DPH)_2_ in Toluene. a) fsTA spectra at intervals across the measurement window for (*m*DPH)_2_. b) Corresponding nsTA spectra. c) Normalized kinetics from both fsTA (circles) and nsTA (crosses) experiments in the regions of the main triplet-pair PIA peak (430–450 nm) and the NIR feature (730–750 nm). The nsTA and fsTA data were stitched by multiplying the nsTA data by appropriate prefactors. d) Time-correlated single photon counting emission lifetime plot of (*m*DPH)_2_, with emission lifetime components and also the PLQE indicated.

At early time scales, ≲1 ps, the fsTA spectra of the dimer, (*m*DPH)_2_, exhibits the characteristic form of a DPH singlet state, S* (Figure 3a). We note that S* is typically utilized to denote the singlet state in the DPH SF literature,^30,31^ since the S_1_ and S_2_ states are close in energy and believed to rapidly equilibrate on subpicosecond time scales.^32−35^ The S* fsTA spectrum is consistent with previously reported DPH transient absorption spectra^29,36^ and comprises of a relatively broad photoinduced absorption (PIA) feature from 3.1–2.2 eV (400–560 nm) and an additional PIA feature in the near-infrared (NIR) region from 1.8–1.6 eV (690–770 nm).

Within 100 ps, the spectrum evolves with the growth of an intense new PIA centered at ∼2.8 eV (∼440 nm). This feature can be attributed to the formation of ^1^(TT),^29^ and may be referred to as the triplet-pair PIA.

Concomitant with the growth of the triplet-pair PIA feature, the NIR PIA of the singlet decays in intensity, although not completely to zero. Instead, this NIR PIA plateaus at ∼30% of its maximum intensity. This result suggests that an equilibrium, with both forward and backward rates on the order of tens of picoseconds, is established between S* and ^1^(TT), such that a moderate singlet population is retained at peak triplet-pair population. Fitting of the data to such an equilibrium model finds time constants of 18 and 100 ps for the forward and reverse processes respectively (SI Figure S2), from which the singlet fission yield, Φ_TT_, may be determined to be 85%.

On the time scale of the nsTA Instruments response (∼1–2 ns), the nsTA spectrum of (*m*DPH)_2_ has the same features as the latter interval fsTA spectra (Figure 3b), albeit with modest differences that can be attributed to the different probe characteristics of the two TA setups. Nevertheless, a dominant triplet-pair PIA peaks at 2.8 eV (440 nm) and there is a weaker PIA feature in the red-NIR spectral region.

At very long delay intervals, from ∼100 ns, there is weak residual intensity in the region 3.0–2.6 eV (410–480 nm). The weak 3.0–2.6 eV PIA has a slight hypsochromic shift vs the triplet-pair PIA and is a close match to the sensitized triplet spectrum of the material (SI Figure S3b). Additionally, the lifetime of this feature is ∼30 μs and is consistent with the lifetime of an isolated DPH triplet state. It is evident from the intensity of the long-lived triplet feature versus the triplet-pair peak that the yield of isolated triplets is very low.

Both the main triplet-pair PIA and residual NIR feature decay on nanosecond time scales (Figure 3b–d). Moreover, fluorescence experiments indicate that the eventual decay of the entire triplet-pair population is mediated by reformation of S* via the S* ⇌ ^1^(TT) equilibrium. To elaborate, we now consider the emissive properties of (*m*DPH)_2_. First, we note that the form of the photoluminescence spectrum is typical of DPH singlet emission (Figure 2) and as such the pathway for emission appears to be via reformation of the singlet. Second, the photoluminescence quantum yield (PLQE) of (*m*DPH)_2_ is 73%, a value that is comparable to the intrinsic PLQE of the DPH chromophore unit.^29,32,33^ This suggests that while triplet-pair states are readily generated in (*m*DPH)_2_, that the decay of the excited state population is ultimately mediated by the repopulated singlet state, S*. Moreover, the small-long-lived isolated triplet population seen in nsTA can be rationalized by S* → T_1_ intersystem crossing (ISC) acting as a minor S* decay pathway that is weakly competitive with fluorescence; ISC is known to act as a minor decay pathway in monomeric DPH materials that similarly decay via the DPH singlet state.^29,37^

The observed singlet mediated excited state decay can only be possible if all transitions involving the triplet-pair states are reversible such that these states interconvert with the singlet in dynamic equilibrium. Direct losses from the triplet pair states, such as internal conversion from ^1^(TT) to the ground state, must be minimal.

The photoluminescence decay appears biexponential with two characteristic lifetime components (Figure 3d). The faster fluorescence component, 4.4 ns, is of a comparable time scale to the intrinsic fluorescence lifetime of the DPH chromophore, as determined by the study of monomeric DPH derivatives.^29,32,33,38^ Meanwhile, the dominant photoluminescence lifetime component, of 13.1 ns, is somewhat longer, demonstrating the capacity for the equilibrium to bias the excited state population away from S*.

### Transient Absorption Spectra of DPH Trimers

Triplet-pair formation in the trimers was also investigated using fsTA. The fsTA spectra of both trimers are almost identical to those of the dimer, with closely matching kinetics for the rise of the triplet-pair signal and decay of the NIR PIA (SI Figure S4). These results indicate that the additional DPH unit in each of the trimers does not strongly influence the initial singlet fission step, i.e. formation of ^1^(TT) from S*. It can be inferred that ^1^(TT) generation occurs on adjacent chromophore units within the trimeric materials and the singlet fission yield of Φ_TT_ = 85%, determined for the dimer, may also be assigned to each trimer.

**Figure 4:**
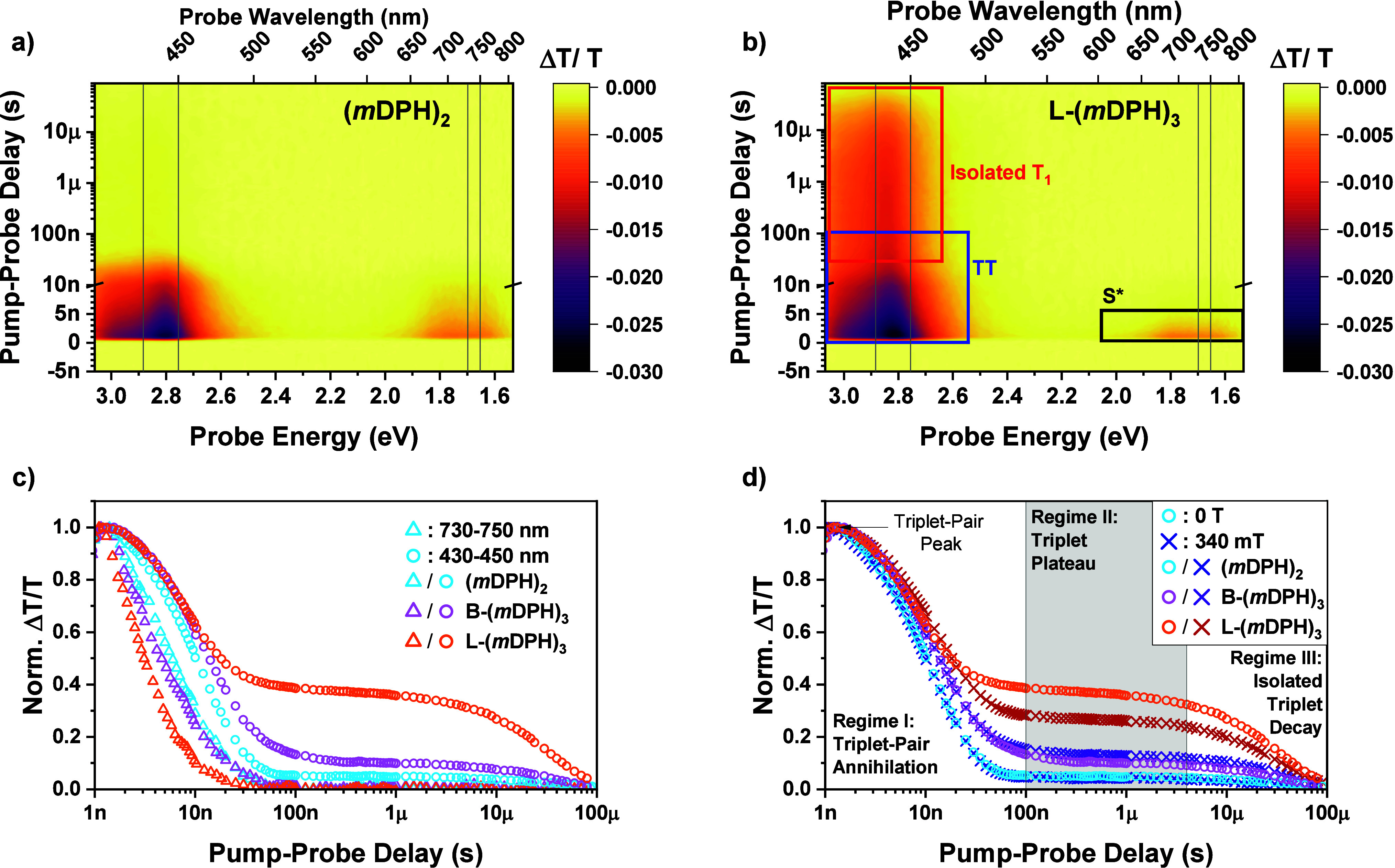
Nanosecond Transient Absorption Comparison of the Trimers to the Dimer. a) nsTA contour map for (*m*DPH)_2_. b) nsTA contour map for L-(*m*DPH)_3_, with key PIA features labeled by their assignment. c) Normalized nsTA kinetics for all three materials in both the NIR region (triangles|730–750 nm) and triplet/triplet-pair region (circles|430–450 nm), corresponding to the regions between the gray vertical reference lines in the contour plots. d) Triplet region nsTA kinetics as in c) with the addition of the corresponding kinetics when the TA measurements were carried out in a magnetic field of 340 mT ± 40 mT.

Consistent with the fsTA results, at the initial instrument response limited nsTA intervals both trimers exhibit similar spectra to the dimer. The spectra of all three materials feature a strong triplet-pair PIA and weaker residual NIR singlet signal (Figure 4a–b and SI Figure S5). However, there are major divergences in the nsTA decay kinetics of the key spectral features.

In (*m*DPH)_2_ the main triplet-pair feature decays to leave a very small persistent PIA with lifetime of approximately ∼30 μs and ∼5% of the intensity of the peak triplet-pair PIA. As discussed above, this persistent PIA can be assigned to a small population of isolated triplet states. These are generated by ISC from the singlet, which must be repopulated by triplet-pair annihilation in the dimer.

In both trimers, the ratio of long-lived triplet PIA intensity relative to the initial triplet-pair PIA peak is enhanced versus the dimer. This is clearly seen as a raised plateau in the kinetics of the triplet/triplet-pair PIA region at 430–450 nm (Figure 4c-d). We will refer to this feature as the “triplet plateau”. In the branched trimer this enhancement of the triplet plateau is modest, with a plateau height ∼10% of the triplet-pair peak. In the linear analogue, the enhancement is much more pronounced with the plateau occurring at ∼40% of the peak PIA intensity.

Consideration of normalized spectra shows that, as with the dimer, that the long-lived triplet PIA has a slight hypsochromic shift vs the main triplet-pair PIA and is a closer match to the sensitized triplet spectrum (SI Figure S3). Taking this in conjunction with the lifetime of the triplet plateau being consistent with isolated triplets and a comparison between the behavior of the materials in toluene versus solid-state solution in polystyrene (SI Figure S6), we assign the long-lived triplet populations to molecules that no longer support a pair of triplet states but only a single isolated triplet.

Despite the increase in triplet plateau height there are only modest differences between the primary rates of triplet-pair annihilation of the materials, as seen from the time scale of decay from the triplet-pair peak to the triplet plateau (Figure 4c–d). There is, however, a clear parallel trend in the rate of decay of the NIR singlet feature, as monitored by the kinetics from 730–750 nm (Figure 4c). Increased triplet-plateau height is accompanied by faster quenching of the singlet population.

As discussed for the dimer, the residual singlet feature remains on nanosecond time scales due to incomplete conversion of S* to ^1^(TT). The decay of this singlet population may proceed via one of two mechanisms: straightforward fluorescence decay from the S* population, or a change in the nature of the triplet pair that reduces its coupling to the singlet. The intrinsic radiative rate of S* is anticipated to be similar in all three materials, being primarily determined by the intrinsic radiative rate of the base DPH chromophore. Consequently, the trend in singlet quenching is suggestive that the rate of triplet-pair spin evolution from ^1^(TT) increases when going from (*m*DPH)_2_ → B-(*m*DPH)_3_ → L-(*m*DPH)_3_.

The critical difference between the dimer and trimeric materials is that in the dimer any formed triplet-pair is trapped on adjacent chromophore sites. This can be anticipated to inhibit significant reductions in the intertriplet coupling. In each trimer there is a possibility that the adjacent triplet-pair may separate via triplet hopping from the central chromophore to the third DPH chromophore unit.^39,40^ Hopping would generate a spatially separated triplet-pair, in which the triplets are localized on nonadjacent DPH units. Upon such separation, the exchange interaction between the triplets must necessarily be significantly weakened versus the adjacent triplet-pair. Thus, the spatially separated triplet pair states can be anticipated to be weakly exchange-coupled, i.e. of (T···T)^*l*^ rather than ^M^(TT) nature. The enhancement of the triplet plateau in the trimers, corresponding to an increased formation of isolated triplets, is evidence for the capacity for weakly exchange coupled triplet pairs to be formed in those materials. This is because weakly exchange coupled triplet pair states may mediate an alternative mechanism for the formation of isolated triplets: one that is more effective than the mechanism possible in the dimer, namely ISC from S*.

Upon triplet-pair separation of ^1^(TT), only the subset of (T···T)^*l*^ states that possess overall singlet character may be initially populated.^21^ Due to symmetry considerations these initially accessible states have mixed singlet-quintet character but no overall ^3^(TT) character, while other states in the manifold may possess mixed quintet-triplet nature.^14,41−44^ However, once some subset of the (T···T)^*l*^ manifold is populated, spin relaxation results in population of the remaining (T···T)^*l*^ states,^25,26^ as the system tends toward an even distribution across the whole (T···T)^*l*^ manifold at thermal equilibrium.

Following separation of ^1^(TT) to access the (T···T)^*l*^ manifold, a subsequent significant increase in *J*_*ex*_ must force the system to collapse into one of the pure-spin ^M^(TT) states. This process, the opposite of triplet-pair separation, is geminate triplet fusion. The probability of forming each pure-spin state of multiplicity M will be directly dependent on the character of that pure-spin state that the occupied (T···T)^*l*^ state possesses at the instant it undergoes fusion. Thus, the consequences of geminate triplet pair fusion must be strongly dependent upon the efficiency of spin relaxation in the weak exchange regime.

If triplet hopping occurs from (T···T)^*l*^ such that ^1^(TT) is reformed, the system has reverted to the state initially produced by SF and, in the absence of a further separation event, this might lead to excited state decay via the singlet manifold. Meanwhile, the formation of ^5^(TT) is strictly reversible due to the spin-forbidden nature of direct deactivation to the ground state and the lack of any energetically accessible single chromophore quintet states to mediate deactivation.^45^ The same is not true for ^3^(TT). Single chromophore higher excited triplet states, T_n_, particularly T_2_, can have energy similar to or less than the energy of S_1_ and ^M^(TT).^21,46,47^ Where ^3^(TT) is close in energy to a T_n_ state, internal conversion (IC) can lead to ultrafast deactivation of ^3^(TT) through the triplet manifold to generate a single isolated triplet, i.e. “T_1_ + S_0_”.^21,48−50^ In fact, even where T_2_ is theoretically energetically inaccessible, as in tetracene and pentacene derivatives, a vibrationally excited T_1_ state has been proposed to facilitate direct IC from ^3^(TT) → T_1_.^51^

Hence, the observed magnitude of the triplet plateau in L-(*m*DPH)_3_ and B-(*m*DPH)_3_, exceeding that which can reasonably be produced by ISC from S*, is indicative of the occurrence of the ^3^(TT) mediated pathway for the formation of T_1_. By extension, this requires that these materials are, to some degree, capable of the generation of weakly interacting triplet-pairs that are sufficiently persistent for spin relaxation to precede geminate fusion.

Moreover, the interaction of any spatially separated triplets can be anticipated to be strongly influenced by the connectivity between the terminal chromophore units and thus be strongly dependent upon the overall trimer geometry. The difference in triplet plateau height, arising from the production of isolated triplets, indicates that the formation of ^3^(TT) occurs much more readily in L-(*m*DPH)_3_ than in B-(*m*DPH)_3_. This suggests a greater barrier to spin relaxation within the (T···T)^*l*^ manifold of B-(*m*DPH)_3_. This may arise from greater residual intertriplet coupling of the separated triplet-pair states in B-(*m*DPH)_3_ than in L-(*m*DPH)_3_. Greater coupling in the branched configuration can be rationalized for the following reasons. First, the branched configuration results in the terminal DPH chromophores being closer in spatial proximity, which could have an effect if there is any through-space contribution to coupling. Second, significantly fewer bonds separate the terminal chromophores across the central DPH unit: 2 in the branched configuration vs 11 in the linear arrangement. Third, there is only one *meta*-phenylene ring in the conjugative pathway between the separated triplets in the branched configuration but two in the linear arrangement; each *meta*-phenylene may be anticipated to increase the degree of destructive quantum interference between the separated triplets.^52^

The proposed origin of the enhanced triplet plateau corresponds to a nonradiative decay pathway that competes with the radiative S* mediated decay pathway that dominates for (*m*DPH)_2_. Consequently, the trend in triplet plateau height would be expected to be accompanied by a trend in decreasing PLQE of the materials. This is observed, with L-(*m*DPH)_3_ and B-(*m*DPH)_3_ having PLQEs of 21% and 54%, respectively, in comparison to 73% for (*m*DPH)_2_.

### Magnetic Field Effects and Fluorescence Decays

Magnetic field effects on singlet fission have been studied by a number of authors,^25,40,44,53−58^ albeit with only a handful of such studies pertaining to iSF materials to the knowledge of the present authors.^40,44,59,60^

To gain further insight, additional nsTA measurements were carried out on our materials in the presence of an external magnetic field (Figure 4d). The decay kinetics of the dimer showed no change. However, magnetic field effects (MFEs) were observed on the nsTA kinetics of both trimers.

In L-(*m*DPH)_3_ there is considerable suppression of the triplet plateau upon application of a magnetic field. Additionally, there is a marginal increase in the triplet-pair lifetime as seen from a slightly slower decay to produce the triplet plateau. Meanwhile, in B-(*m*DPH)_3_ the magnitude of the MFE, like the magnitude of the triplet plateau vs the dimer, is smaller than in its linear analogue. More intriguingly, the direction of the MFE is inverted such that the triplet plateau height is slightly enhanced in the presence of a magnetic field.

**Figure 5:**
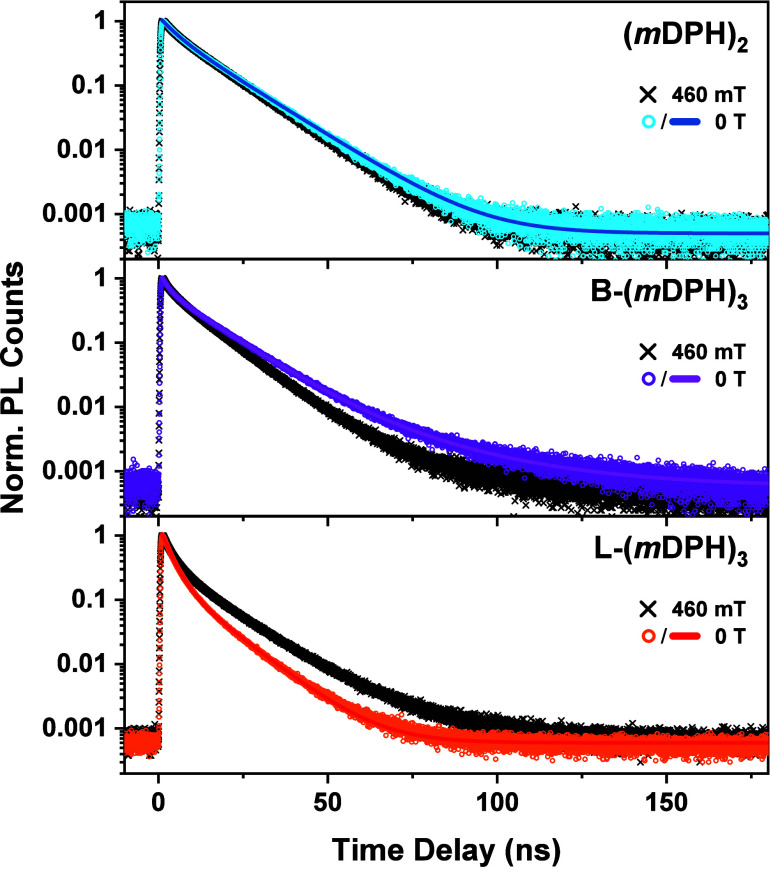
Magnetic field Effects on Fluorescence Decay. TCSPC emission lifetime plots (exc. 375 nm) of all three materials. Colored circles indicate data recorded in the absence of a magnetic field, while black crosses indicate data recorded with the sample positioned between two magnets with a central field strength of 460 ± 30 mT. Colored lines indicate multiexponential fits to the zero-field data. Lifetime parameters from these fits are tabulated in Table 1.

To support the MFE results obtained for the TA experiments, further magnetic field dependence studies were undertaken on the photoluminescence decay of the materials. TCSPC was utilized to investigate the fluorescence decay in the absence and presence of an external magnetic field (Figure 5 and Table 1).

**Table 1:** Fluorescence yields and lifetimes in zero field

**Compound**	PLQE/%	**τ (τ**_**1**_**; τ**_**2**_**; τ**_**3**_**)/ns**	**Relative Amplitudes (A**_**1**_**; A**_**2**_**; A**_**3**_**)**
**(*****m*****DPH)**_**2**_	73	4.4; 13.6	0.36; 0.64
**B-(*****m*****DPH)**_**2**_	54	2.3; 12.2; 30	0.45; 0.53; 0.02
**L-(*****m*****DPH)**_**2**_	21	2.9; 10.6	0.82; 0.18

The fluorescence decay profile of (*m*DPH)_2_ is as discussed above and is found not to be perturbed by application of a magnetic field.

In L-(*m*DPH)_3_ the fluorescence decay curve is noticeably distinct from that of the dimer with an overall increased rate of decay. Like (*m*DPH)_2_ the fluorescence decay of L-(*m*DPH)_3_ was described by a biexponential function, albeit with a much-reduced fraction of delayed fluorescence compared to the initial prompt fluorescence and shorter associated time constants. The increase in prompt decay rate supports the interpretation that intramolecular triplet separation expedites the loss of singlet character of the initially formed triplet-pair in L-(*m*DPH)_3_ compared to (*m*DPH)_2_. Furthermore, the reduced fraction of delayed fluorescence vs (*m*DPH)_2_ highlights that triplet-pair evolution in L-(*m*DPH)_3_ is less reversible than in the dimer, resulting in additional nonradiative decay.

Upon application of a magnetic field, a significant MFE is observed in L-(*m*DPH)_3_ wherein the PL is increased at delayed time scales.

Lastly, the fluorescence decay profile of B-(*m*DPH)_3_ also features faster initial prompt decay than in the dimer but additionally required a weak tertiary delayed fluorescence component to fully describe the decay. Perhaps most significantly, this material exhibits suppressed fluorescence at longer time scales when a magnetic field is applied. Such an MFE is inverted relative to the MFE in L-(*m*DPH)_3_ and is consistent with the inverted MFE observed on the nsTA kinetics.

## Discussion

It is well established that MFEs in SF and TTA-UC systems arise from the modulation of triplet-pair states in the presence of a magnetic field. In the originally formulated Johnson-Merrifield model^41,61−63^ and later adaptations,^25,53^ MFEs are attributed to a change in the number of weakly coupled triplet-pair states that have singlet character when a magnetic field is applied. When considered in this way, MFEs formally arise from changes in the rates of singlet fission and triplet fusion processes. More recently, Yago, Wakasa, and co-workers have developed an alternative model for MFEs in singlet fission that they termed the triplet-pair model,^55^ but which may be more unambiguously referred to as the Yago-Wakasa model. The Yago-Wakasa model incorporates influences from the exchange interaction and spin coherences that are either not at all or are poorly treated in the Johnson-Merrifield model. Primarily, this has facilitated the interpretation of high-field effects arising from level-crossing interconversions of strongly exchange-coupled pure-spin triplet-pairs in strong magnetic fields exceeding 1 T.^54,55,64^ In the Yago-Wakasa model, at low magnetic fields (<1T) MFEs arise from modulation of the coherent conversions between states in the triplet-pair manifold,^55^ as opposed to the rate with which S_1_ interconverts with that manifold, as under the Johnson-Merrifield model. Nevertheless, under either model, only weakly exchange-coupled triplet-pairs can be influenced by an external magnetic field of low strength (≲1 T). Therefore, the existence or lack thereof of MFEs provides insight into the capacity of a singlet fission system to form separated triplet-pairs with weak exchange-coupling.

For the trimers studied in this work, the MFEs can be rationalized by perturbation of the weakly exchange-coupled triplet pairs that originate from spatial separation of the triplets onto nonadjacent chromophores. For both trimers, the onset of MFEs appears to occur after several nanoseconds, indicating that triplet separation only occurs on these time scales. Furthermore, while magnetic fields influence the fluorescence decay and formation of the nsTA triplet plateau, there is not a significant impact on the decay of the triplet plateaus themselves (Supporting Information Figure S7). This supports the assertion that by the point that the plateau has been established there is minimal remaining triplet-pair population. The isolated triplets to which the long-lived triplet PIA is attributed are not strongly perturbed by a magnetic field. This is further evidence that the triplet plateau predominantly originates from isolated triplets rather than long-lived pairs of triplets since application of a magnetic field would be anticipated to modulate triplet–triplet annihilation of the latter.

The MFEs on the fluorescence decays are intuitively consistent with the MFEs observed on the nsTA kinetics. For L-(*m*DPH)_3_, upon application of a magnetic field, increased photoluminescence on delayed time scales indicates that a greater proportion of separated triplet-pairs reform ^1^(TT) and decay radiatively via S* than in the zero-field case. Consequently, fewer separated triplet-pairs can decay via the ^3^(TT) mediated pathway and the yield of persistent isolated triplets is reduced relative to the zero-field case. Meanwhile, for B-(*m*DPH)_3_ the magnetic field increases the fraction of the overall triplet-pair decay that proceeds via the triplet manifold. Consequently, the radiative outcoupling of separated triplet pairs via ^1^(TT) is reduced. We note that the structural dependence of the sign of MFEs is not so dissimilar to results previously observed in tetracene derived iSF materials. The behavior of L-(*m*DPH)_3_ is akin to the magnetic field dependent fluorescence properties of the tetracene trimer and tetramer, linked via the 5-position with *para*-phenylenes, that were studied by Wang et al.^40^ Meanwhile, the direction of MFEs observed in B-(*m*DPH)_3_ is instead reminiscent of the MFEs that were recently observed by Kim et al. in a TIPS-tetracene hexamer and dimer with connectivity to the respective linkers via the 2-position.^59^

The differences in the PLQEs, the signs of the MFEs, and the triplet plateau heights of L-(*m*DPH)_3_ and B-(*m*DPH)_3_ highlight that the triplet–triplet arrangement remains important even for separated triplet-pair states with weak exchange-coupling. The coupling geometry of (T···T)^*l*^ demonstrates a significant influence on the spin-relaxation process that is required to facilitate ^3^(TT) formation. This manifests as a significant divergence in the balance between the various triplet-pair decay pathways for each of the isomeric trimers. Moreover, the coupling arrangement demonstrably determines whether application of a magnetic field biases the decay toward the ^1^(TT) or ^3^(TT) mediated decay channel. These dependencies on geometry would not be expected if triplet hopping simply yielded the *J*_*Ex*_ → 0 limit of noninteracting triplets and highlight the critical need to distinguish weakly coupled triplet pairs from “free triplets”. This distinction is obviously most essential in bounded systems but should remain relevant in unbounded systems wherein the weak exchange and zero interaction regimes are both possible.

By comparison to the trimeric materials, the combined lack of a significant triplet plateau or MFEs in (*m*DPH)_2_ appears to indicate that this material is incapable of forming weakly coupled triplet pairs i.e. that the triplet-pair population remains bound in the strongly coupled ^1^(TT) state. Without intermediary mixed-spin (T···T)^*l*^ states, there is no facile pathway by which ^3^(TT) can form and mediate the decay to “T_1_ + S_0_”.

**Figure 6:**
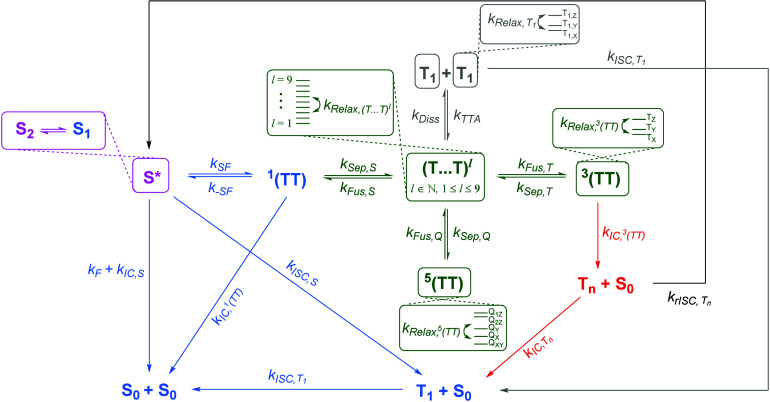
A general model for singlet fission (SF) or triplet–triplet annihilation upconversion (TTA-UC). Different colors emphasize which aspects of the model are applicable in different materials systems. The part in pink is specific to materials based upon diphenylhexatriene (DPH) or possibly other oligoenes or polyenes, such as carotenoids. In blue is the portion of the mechanism that corresponds to SF in a strongly constrained system, wherein only the strong exchange regime is accessible; this is considered to be the case for (*m*DPH)_2_. The part in green and red is relevant to larger bounded systems, wherein weakly exchange coupled triplet pairs can be formed; this is considered to be applicable to certain larger covalent oligomers, including L-(*m*DPH)_3_ and B-(*m*-DPH)_3_. The part in black represents a high-level reverse intersystem crossing process that repopulates the singlet from T_n_; this has been shown to be significant in some specific materials such as rubrene,^21^ but has not yet been either demonstrated or conclusively ruled out in many materials, including our intramolecular DPH derivatives. Lastly, the portion of the mechanism in gray is only applicable to the case of unbounded systems, such as either solid-state materials or solutions for intermolecular SF or TTA-UC. It should be noted that the sublevels of the T_1_, ^3^(TT), and ^5^(TT) state manifolds are labeled with Cartesian axes and are representative of the zero-field scenario; in the presence of an external magnetic field, these are perturbed by the Zeeman effect and labels such as Q_+2_, Q_+1_, Q_0_, Q_–1_, Q_–2_, T_+1_, T_0_, and T_–1_ should be applied.

To summarize our results, we present a general model for SF/TTA-UC in a form that is more complete than shown throughout the literature (Figure 6). This aims to clearly represent the different exchange coupling regimes and highlight the manifolds within which spin relaxation can occur. The subset of the model that pertains to the bounded DPH oligomers systems discussed in the present manuscript is emphasized in color. The model may be generally applicable to any SF or TTA-UC system and is adaptable to both unbounded systems and bounded systems for which the free triplet limit is unachievable. In regard to the general accessibility of the weak-exchange regime in iSF materials, we note that the general ability to form weakly exchange-coupled triplet pairs is not as straightforward as the dichotomy between dimers and larger oligomers. While this dichotomy holds for the materials discussed in the present manuscript, the failure of triplet-pair separation to occur has been reported in several other oligomeric iSF systems.^36,67,68^ Meanwhile, dimers with greater conformational freedom than (*m*DPH)_2_ can be capable of sufficient *J*_*Ex*_ reduction to access the weakly coupled regime.^43,44,69,70^

## Conclusions and Outlook

A contiguously connected dimer of the singlet fission active chromophore DPH has been synthesized. This material, (*m*DPH)_2_, demonstrates significant, albeit incomplete, triplet-pair generation within 100 ps. A lack of magnetic field effects in this material disbars the formation of significant populations of weakly exchange-coupled triplet-pair states, (T···T)^*l*^. Moreover, the comparability of the PLQE of this material to monomeric DPH derivatives requires that the excited state decay is dominated by reformation of the singlet, S*; for this particular dimer other losses such as direct nonradiative deactivation from ^1^(TT) must be minimal.

Derivative trimers based upon the DPH-DPH connectivity of (*m*DPH)_2_ were additionally synthesized, featuring branched and linear configurations. The additional chromophore site in both trimers presents the capacity for the formation of weakly exchange-coupled triplet-pairs by triplet hopping. Via spin-relaxation within the (T···T)^*l*^ manifold, this facilitates an alternative triplet-pair decay pathway that results in the generation of a single isolated triplet. The balance between the different triplet-pair decay pathways is strongly dependent upon the geometry of the weakly coupled triplet-pair state and can be perturbed by alteration of the mixing of the (T···T)^*l*^ states by a magnetic field. Magnetic field effects represent a historically underused probe for the study of intramolecular singlet fission materials. Through purely qualitative interpretation of MFEs on the TCSPC and TA experiments, we have elucidated the capacity for weakly exchange-coupled triplet-pair states to be formed in the reported materials. In future work, this could be extended to a quantitative treatment using the theoretical framework of the Yago-Wakasa model.^55^

Finally, by consideration of our results in the context of the wider SF and TTA-UC literature we have presented a schematic model for the SF and TTA-UC processes that is more complete than any previously depicted. This should present a valuable starting point for the interpretation of SF and TTA-UC materials.

## Supporting Information Available

Experimental details including synthetic procedures, additional spectroscopic results, and NMR spectra (PDF)
