# Supplementary material for: The Interplay of Strongly and Weakly Exchange-Coupled Triplet Pairs in Intramolecular Singlet Fission
Source: J Am Chem Soc. 2024 Oct 17;146(43):29664–74. doi: 10.1021/jacs.4c10483 (PMC11528409; doi:10.1021/jacs.4c10483)
Supplement: Supplementary file 1 — ja4c10483_si_001.pdf [file ja4c10483_si_001.pdf]

Supporting Information

The interplay of strongly and weakly exchange-coupled triplet-pairs in intramolecular singlet fission

Oliver Millington†‡§, Stephanie Montanaro†§, Ashish Sharma‡, Simon A. Dowland‡, Jurjen Winkel‡, Jeannine Grüne‡, Anastasia Leventis†, Troy Bennett†, Jordan Shaikh‡, Neil Greenham‡, Akshay Rao*‡, Hugo Bronstein*†‡

† Department of Chemistry, University of Cambridge, Cambridge, CB2 1EW, UK.

‡ Cavendish Laboratory, University of Cambridge, Cambridge, CB3 0HE, UK.

Table of Contents

1. **EXPERIMENTAL DETAILS** **2**

2. **FURTHER TRANSIENT ABSORPTION SPECTROSCOPY** **3**

   a) Kinetic Modelling for (mDPH)$_2$ *3*
   b) Comparison of Long-lived TA Signal to Sensitized Triplet Spectrum for (mDPH)$_2$ *5*
   c) fsTA Spectra and Kinetics of All Three Materials *6*
   d) nsTA Contour Plot of B-(mDPH)$_3$ vs (mDPH)$_2$ *6*
   e) nsTA Triplet Kinetics in Toluene Solution versus a Rigid Polystyrene Matrix *7*
   f) MFE on nsTA Kinetics for Trimers Normalized at 10 µs *9*
   g) Trimer nsTA Signal Decay Fitting and Note on Minor Contribution from Pairs of Triplets *10*

3. **SYNTHETIC INFORMATION** **11**

4. **NMR SPECTRA** **15**

5. **MASS SPECTRA OF OLIGOMERS** **21**

6. **REFERENCES** **22**

1. Experimental Details

General experimental details for synthesis, synthetic characterisation, (zero-field) transient absorption spectroscopy, (zero-field) time-correlated single photon counting, photoluminescence quantum yield, steady-state absorption, and steady-state photoluminescence were as reported in detail in the supplementary information of our previous work.[1] In some of the supplementary results included herein, data for a monomeric reference compound, *p*Tol-*m*DPH, is included for comparison. The synthesis of this material and its photophysical behaviour have also been previously reported in detail.[1]

Magnetic Field Dependant TA and TCSPC Measurements

TA: The nsTA experiments with a magnetic field were conducted using a pair of neodymium magnets. The magnets are housed in a custom 3D printed cuboid that features a central slot so that a cuvette can be inserted between the magnets. This could be mounted in place of the usual sample holder for TA measurements (Figure S1a). The cuboid has holes in the centre of the four sides so that laser beams can be aligned through the sample. The magnetic field strength in the sample slot was measured to be 340 ± 40 mT, as measured using a Hirst Magnetics GM08 Gauss Meter. The error is indicative of the range of field strengths achievable for different positions of the Gaussmeter probe, within the limits of the volume of space that a beam can pass through the sample slot. Having already aligned the pump-probe overlap for the normal sample holder, overlap on the sample was achieved by optimizing the position of the magnetic sample holder without any further adjustment to the beam paths.

TCSPC: Similarly, magnetic field dependent TCSPC measurements were conducted by replacing the TCSPC sample holder with a cuboidal sample holder housing two neodymium magnets similar to that used for the nsTA measurements. However, this cuboid featured a sample slot angled at 45° to the cuboid faces rather than parallel to them, making it better suited to the geometry of the TCSPC setup. The magnetic field strength in the sample position was measured to be 460 ± 30 mT.

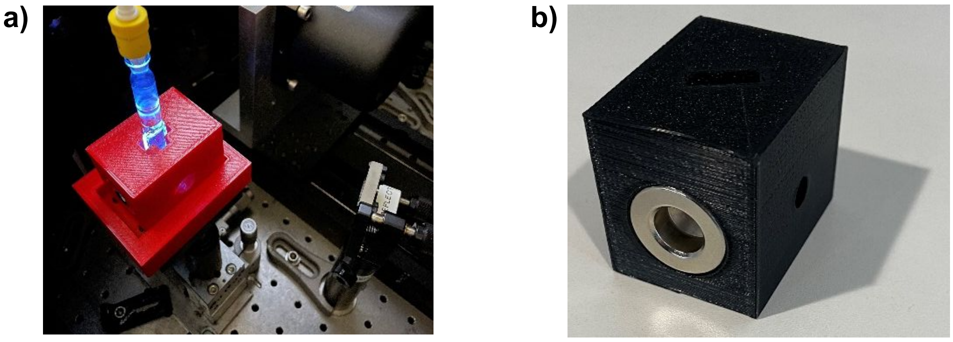

Figure S1. Magnets used for magnetic field dependence measurements. a) Magnetic sample holder with field strength of 340 ± 40 mT in position in the setup used for nsTA measurements. b) The magnetic sample holder used for mag-TCSPC with a central field strength of 460 ± 30 mT and a sample slot at 45° to the sides of the cuboid.

2. Further Transient Absorption Spectroscopy

a) Kinetic Modelling for (mDPH)$_2$

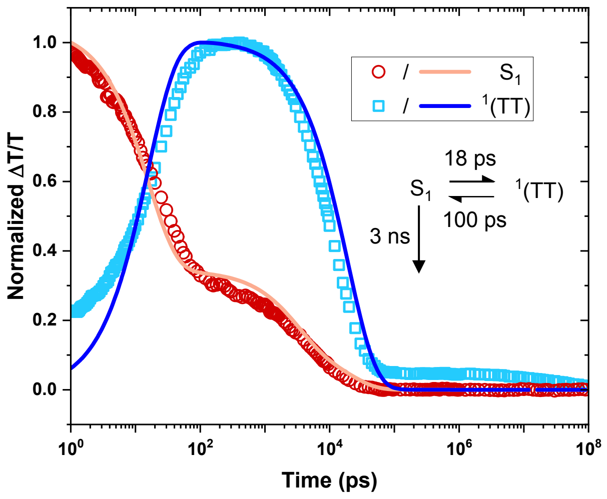

Figure S2. Comparison of simulated kinetics for the dimer, produced using an equilibrium model, and the raw kinetic data for the main spectral features. For simplicity, the model does not include intersystem crossing from S_1 to T_1.

Estimation of Yields of Triplet-Pair States and Isolated Triplets

With the simplifying approximation of ignoring the decay of S_1 to the ground state due to the comparatively slow rate of that process, the rate constants extracted from this model may be utilised to estimate the singlet fission yield, Φ_{TT}, for (mDPH)$_2$ as follows:

$$k_{SF} = \frac{1}{18\text{ ps}} = 0.056\text{ ps}^{-1}$$

$$k_{-SF} = \frac{1}{100\text{ ps}} = 0.010\text{ ps}^{-1}$$

$$\frac{[\text{TT}]_{eq}}{[\text{S}1]_{eq}} = K_{SF} = \frac{k_{SF}}{k_{-SF}} = 5.6$$

$$\Phi_{TT} = \frac{[\text{TT}]_{eq}}{[\text{TT}]_{eq} + [\text{S}_1]_{eq}} = \frac{K_{SF}}{K_{SF} + 1_{SF}} = \frac{5.6}{6.6} = 0.85 = 85\%$$

As discussed in the text, and presented below in SI Figure S4, the kinetics of triplet-pair formation are all but identical for the three materials. The additional DPH units of the trimers do not significantly impact the initial singlet fission process of generating triplet-pair states. Hence, Φ_{TT} = 85% can be treated as the singlet fission yield for all three materials.

The triplet signal seen in TA represents a convoluted mixture of signals from strongly coupled triplet pair states, weakly coupled triplet pair states, and isolated triplets following the annihilation of one triplet per pair. Consequently, it is not possible to directly extract a yield for the spatially separated weakly coupled triplet pairs, $(T\ldots T)^l$, that are subsequently formed in the trimers, following the initial production of strongly coupled TT states.

However, we may determine lower bounds for the yields of $(T\ldots T)^l$ in the trimers by first considering the yields of persistent isolated triplet states. We will start by making the assumption that absorption cross section of a triplet-pair state is equal to the absorption cross section of two triplets. This is equivalent to treating the singlet fission yield of 85% as equivalent to a peak triplet yield of 170%. The yield of persistent isolated triplets may then be determined by comparing the height of the 'triplet plateau' that is associated with those states to the height of the peak of the triplet/triplet-pair PIA signal. The plateau intensities are ~5%, ~10%, and ~40% for $(m\mathrm{DPH})_2$, B-$(m\mathrm{DPH})_3$, and L-$(m\mathrm{DPH})_3$; this would indicate isolated triplet yields of ~8.5%, ~17%, and ~68% respectively.

We now take the value found for the dimer by this method as the baseline triplet production of ISC from S_1. The value of 8.5% is perhaps a little high compared to the ISC yields of 3-5% reported for various DPH materials,[1,2] but is of the correct order of magnitude and should be reasonable enough for the purposes of this approximate estimation.

Subtracting the ISC baseline, we find that the yields of additional isolated triplets generated via the $(T\ldots T)^l$ mediated pathway in the trimers are ~8.5% and ~60% for B-$(m\mathrm{DPH})_3$ and L-$(m\mathrm{DPH})_3$ respectively. Given that each isolated triplet is generated from a weakly coupled $(T\ldots T)^l$ state, this in turn sets lower bounds on $\Phi_{(T\ldots T)}$ of ~8% and ~59.5%. These are lower bounds because any fraction of $(T\ldots T)^l$ that are formed but eventually decay via reformation of the singlet are missed by this approach. The upper bound for $\Phi_{(T\ldots T)}$ is given by $\Phi_{(T\ldots T)} = \Phi_{TT} = 85\%$.

By considering that all excited states must eventually decay via either fluorescence from S_1, triplet generation by either pathway, or internal conversion (IC) to the ground state from one of the excited states with singlet multiplicity, we may find the yields of the latter process by making up the deficit to 100%.

Table S1. Estimated yields of the excited state processes in the dimer and trimers.

**Material**	Φ_{TT} / %	$\Phi_{(T\ldots T)}$ / %	Φ_{ISC} / %	Φ_F/ %	Φ_{IC}/ %
**$(m\mathrm{DPH})_2$**	85	0	8.5	73	19
**B-$(m\mathrm{DPH})_3$**	85	$8.5 \leq \Phi_{(T\ldots T)} \leq 85$	8.5	54	29
**L-$(m\mathrm{DPH})_3$**	85	$60 \leq \Phi_{(T\ldots T)} \leq 85$	8.5	21	11

b) Comparison of Long-lived TA Signal to Sensitized Triplet Spectrum for $(m\mathrm{DPH})_2$

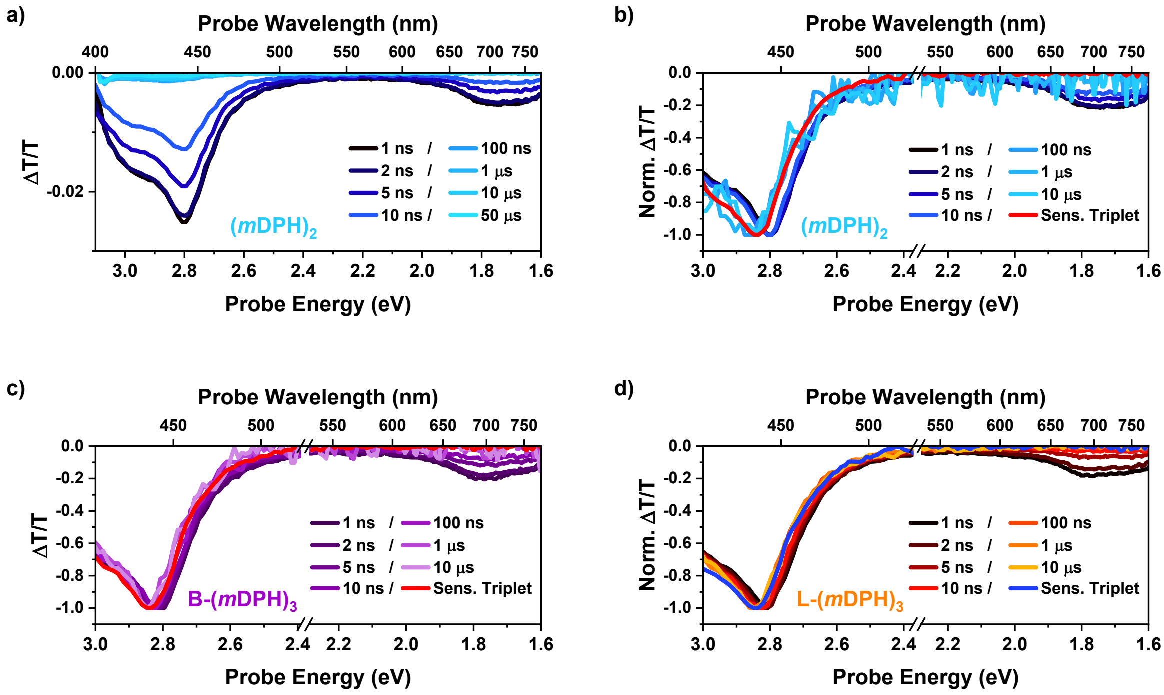

Figure S3. a) nsTA spectra of 1 mM solution of $(m\mathrm{DPH})_2$ in toluene (exc. 400 nm) [Reproduced from Fig 3.b for ease of reference]. b) Normalised spectra from (a), with the sensitized triplet indicated for comparison [500 μM $(m\mathrm{DPH})_2$ exc. 532 nm with 120 μM PdOEP]. A break in the x-axis removes artefacts due to high pump scatter at ~ 532 nm in the sensitization experiment. c) As in (b) but for B-$(m\mathrm{DPH})_3$. d) As in (b) but for L-$(m\mathrm{DPH})_3$.

c) fsTA Spectra and Kinetics of All Three Materials

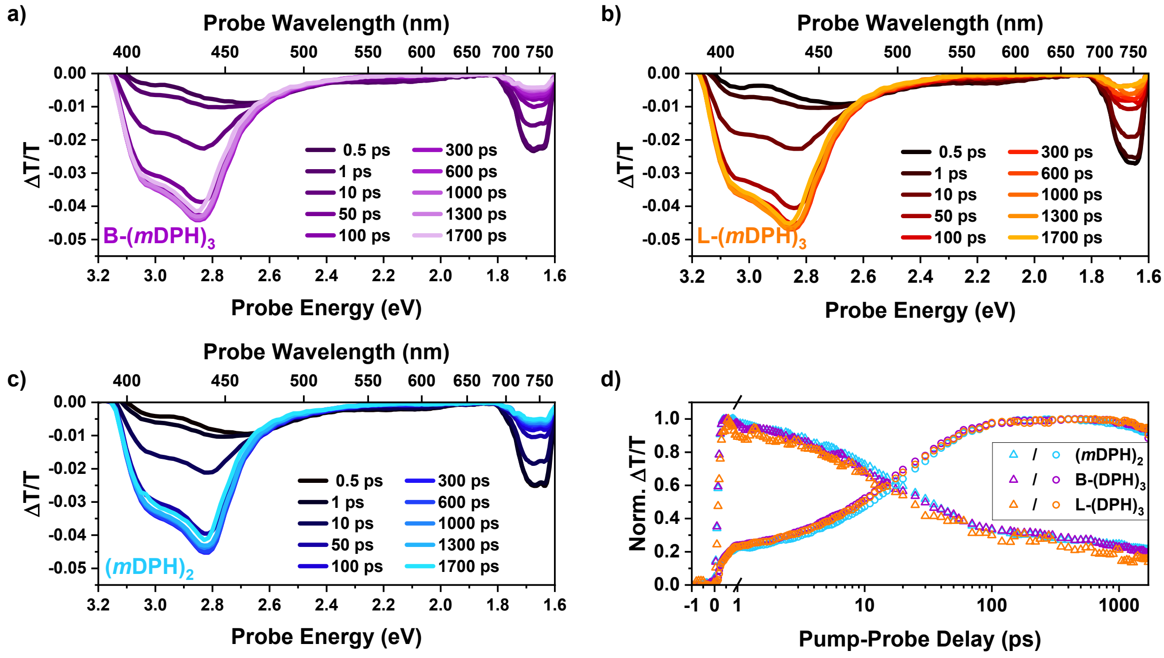

Figure S4. a)–c) fsTA spectra for 1mM solutions in toluene (exc. 400 nm) of: a) B-(mDPH)$_3$, b) L-(mDPH)$_3$, and c) (mDPH)$_2$. d) Normalised fsTA kinetics in the NIR singlet region (triangles|730–750 nm) and the region of the triplet-pair PIA (circles|430– 450 nm).

d) nsTA Contour Plot of B-(mDPH)$_3$ vs (mDPH)$_2$

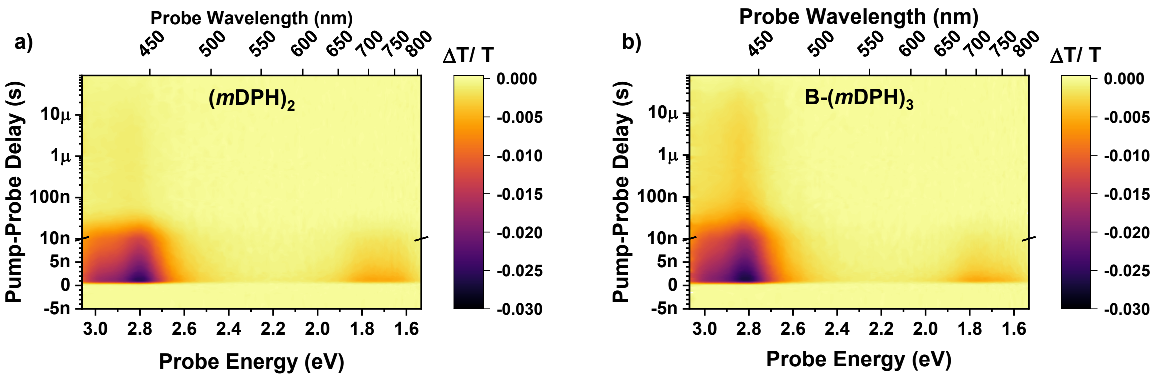

Figure S5. nsTA contour maps for 1mM toluene solutions (exc. 400 nm) of a) (mDPH)$_2$, and b) B-(mDPH)$_3$.

e) nsTA Triplet Kinetics in Toluene Solution versus a Rigid Polystyrene Matrix

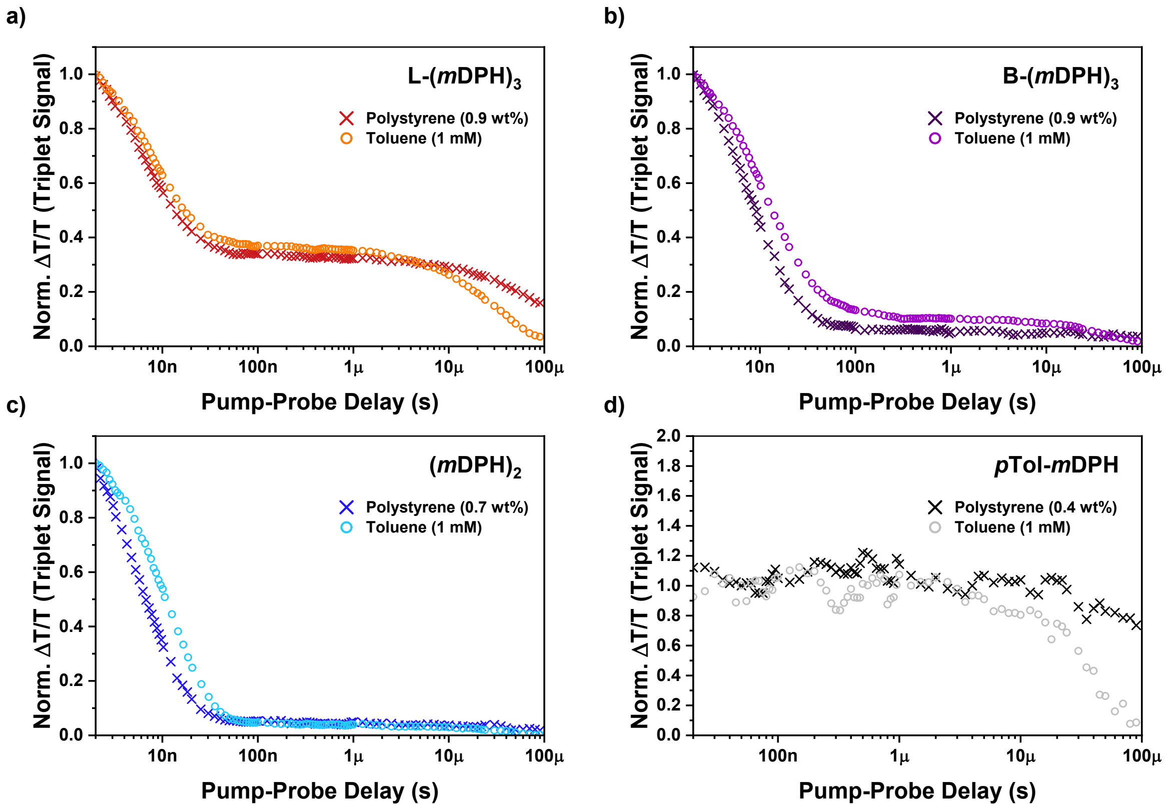

Figure S6. nsTA kinetics of the triplet signal compared between samples made up in toluene solution versus solid-state solutions in a polystyrene matrix (exc. 355 nm). a)-c) Data for the oligomers taken from kinetics integrated over the 430–460 nm wavelength range, normalized at the instrument response limited peak intensity of the triplet-pair PIA. d) Data for a reference monomer, *p*Tol-*m*DPH, taken from the kinetics integrated over the wavelength range 420–440 nm, normalized at 50 ns and showing only time-intervals from 20 ns onwards (after the singlet signal has completely decayed).

Discussion

Throughout the iSF literature, persistent triplet populations have been assigned to "free triplets" *i.e.* "$T_1 + T_1$". For exothermic SF systems, such as those based upon pentacene, such a result is energetically stable relative to S_1 and can readily be rationalized. However, for materials based upon chromophores for which S_1 is lower energy than "$T_1 + T_1$", or is even just readily thermally accessible, the persistence of "$T_1 + T_1$" is counterintuitive. For dimers and oligomers based upon tetracene and perylene, certain authors have rationalized their proposed formation of significant yields of "free triplets" by invoking large conformational changes that purportedly isolate the two triplets.[3–8]

By contrast we have assigned our "triplet plateau" to molecules in which one of the two triplets initially produced by SF has already been lost. Barring minor components that are detailed in SI Section 2g below, the timescale of decay for the triplet plateaus of the oligomers are consistent with the lifetime of

isolated single triplets, as can be seen by comparison to the triplet decay of monomeric reference material *p*Tol-*m*DPH (Figure S6). If instead the plateaus arose from decoupled 'T_1+T_1' arising from conformational changes, then due to the reversibility of such conformational changes, we would expect geminate TTA to lead to the decay of the plateau features. This would have the consequence that the plateaus would decay with a distinct (almost certainly shorter) lifetime from the intrinsic triplet lifetime set by the rate of $T_1 \rightarrow S_0$ intersystem crossing. The fact that we do not observe such a decay, except for the aforementioned minor components, is indicative that the plateaus do indeed correspond to isolated triplets *i.e.* 'T_1+S_0'. Furthermore, the oligomers display negligible fluorescence beyond ~100 ns following photoexcitation (Main Text Figure 5); the decay of the excited states that give rise to to the 'triplet plateau' is non-radiative. But if the plateau did arise from 'T_1+T_1'and decay, at least in part via geminate TTA, then we would expect significant delayed fluorescence on the same timescale as the decay of the triplet plateau.

In light of these arguments, the only way that the plateau features could be ascribed to 'T_1+T_1' rather than 'T_1+S_0' would be if the conformational changes that isolated the triplets were essentially irreversible, *i.e.* reversing more slowly than $T_1 \rightarrow S_0$ intersystem crossing; requiring conformational equilibria so biased that they seem highly unfeasible. Moreover, IF such an irreversible conformational change could occur, said geometric change must necessarily be very significant, the impossibility of which is revealed by considering the behaviour of the materials in polystyrene. In a rigid matrix the capacity for large conformational changes must be strongly inhibited relative to solution in a liquid solvent of low-moderate viscosity (*e.g.* toluene). Thus, if the triplet plateau in L-(*m*DPH)$_3$ did correspond to "T_1+T_1", it should be strongly suppressed in polystyrene relative to toluene due to the inhibition of large conformational changes. However, this is not observed (Figure S6). The relative intensity of the triplet plateau relative to the peak intensity of the triplet-pair PIA is instead very similar for both the solution of L-(*m*DPH)$_3$ in toluene and the solid-state solution in polystyrene.

In (*m*DPH)$_2$ and B-(*m*DPH)$_3$ the rate of triplet-pair annihilation is moderately enhanced in polystyrene; restricting the degree of conformational freedom inhibits the ability for triplet-pairs to lose their singlet character. This also results in a noticeable drop in the triplet-plateau of B-(*m*DPH)$_3$ due to reduced capacity for ^{3}(TT) to form. This suggests a greater significance of conformational changes on the process of spin-evolution in the branched trimer relative to the linear. Critically, this does not mean that in toluene the small plateau for B-(*m*DPH)$_3$ arises from "T_1+T_1" due to irreversible conformational changes. Instead, it is far more feasible that reversible conformational oscillations, causing transient further reductions in J_{Ex}, facilitate the spin relaxation of $(T\ldots T)^l$ that is instrumental in forming "T_1+S_0" via ^{3}(TT).

In all three oligomers the lifetime of isolated triplets is enhanced in the rigid matrix. This can be attributed to an intrinsic enhancement of the DPH triplet lifetime in polystyrene versus toluene, by

comparison to a reference monomeric DPH derivative, *p*Tol-*m*DPH; a rigid matrix suppresses the non-radiative decay of isolated triplet states.

Additional Experimental Information: Preparation of Polystyrene Matrix Samples

In a nitrogen glovebox, a 100 mg/ml solution of polystyrene (M_W ~ 192 kDa, Sigma-Aldrich) was prepared in toluene.1 mM stock solutions of each of the DPH materials were prepared. For each material, a portion of the 1 mM solution was mixed with an equal volume portion of the PS stock solution and mixed thoroughly using a vortex mixer. Making these solutions with stock DPH solutions of equal molar concentration results in polystyrene matrix films that have matching number concentrations of DPH dopant but differing weight percent of DPH dopant, due the differing molecular weights of the materials. The resulting weight percents of DPH oligomer in polystyrene were all less than 1 wt%. Films were prepared by drop-casting the mixed solutions onto glass substrates. These were then allowed to dry overnight in the glovebox. Finally, the dried films were encapsulated using polyisobutylene epoxy to bond a glass coverslip.

f) MFE on nsTA Kinetics for Trimers Normalized at 10 μs

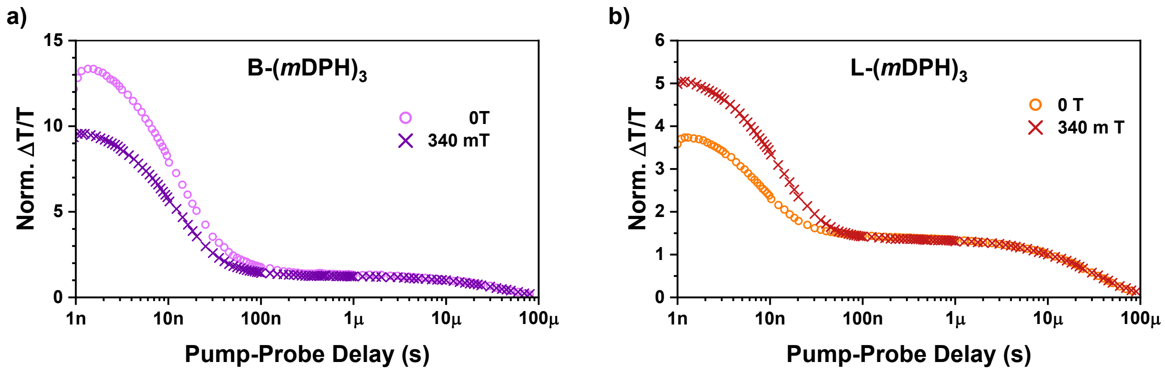

Figure S7. nsTA kinetics for the trimers for the triplet/triplet-pair region (430–450 nm) in the absence and presence of a magnetic field, analogous to the data presented in Figure 2 of the main text but normalized at 10 μs rather than the peak signal. a) B-(*m*DPH)$_3$. b) L-(*m*DPH)$_3$.

By normalizing to the late time period, rather than the early period, it is made clear that the kinetics of the plateau region (≳ 100 ns) are not significantly perturbed by the application of the magnetic field in either trimer.

g) Trimer nsTA Signal Decay Fitting and Note on Minor Contribution from Pairs of Triplets

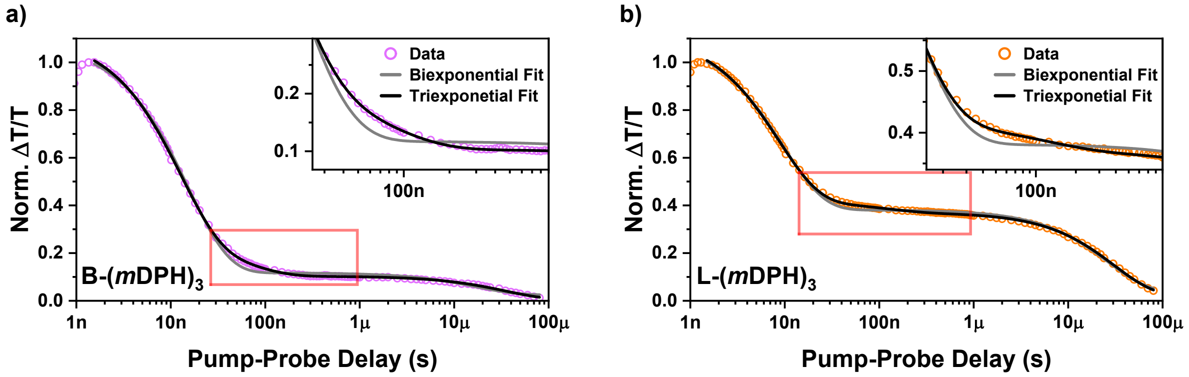

Figure S8. nsTA kinetics for the trimers for the triplet/triplet-pair region (430–450 nm) with comparison of biexponential and triexponential fits to kinetic data. Expansions are indicated for intermediate timescales from 15-1000 ns, for which the incompleteness of the biexponential fits is most obvious. The parameters for the triexponential fits are indicated in Tabe S1. a) B-(*m*DPH)$_3$. b) L-(*m*DPH)$_3$.

The intermediate minor tertiary decay components of the triplet-pair/ triplet decay kinetics of the trimers are indicative that the triplet-pair annihilation processes are inhomogeneous. These intermediate decay components are suggestive of subpopulations of weakly coupled triplet pairs, $(T...T)^l$, that are somewhat more persistent than the majority of $(T...T)^l$ states in each trimer (that decay on timescales of 8-12 ns). Nevertheless, the lifetimes of these somewhat more persistent triplet-pairs (66 ns and 120 ns, respectively) are still orders of magnitude shorter than the lifetimes of singular isolated triplet states (~ 30 μs). The origin of the inhomogeneity may arise from inequivalences in the interconversion rates of certain states in the $(T...T)^l$ manifold or differences in their rates of formation of $^M(TT)$ states that precede annihilation. Given the modest differences observed between toluene solution and polystyrene matrix samples on these early and intermediate timescales (Figure S6), conformational changes may have a role in mediating such transitions.

Table S2. Fitting Paramaters for Decay of the 430-450 nm nsTA Signals (1 mM in toluene, zero-field)

Material	$\tau(\tau_1; \tau_2; \tau_2)$ / ns	Relative Amplitudes (A_1; A_2; A_2)
**(*m*DPH)$_2$**[a]	12; 30000	0.96; 0.04
**B-(*m*DPH)$_3$**[b]	12; 66; 35000	0.77; 0.14; 0.10
**L-(*m*DPH)$_3$**[b]	8.6; 120; 31000	0.60; 0.05; 0.36

[a] Corresponding to fit data presented in Figure 1c.
[b] Corresponding to fit data presented in Figure S8.

3. Synthetic Information

Scheme S1

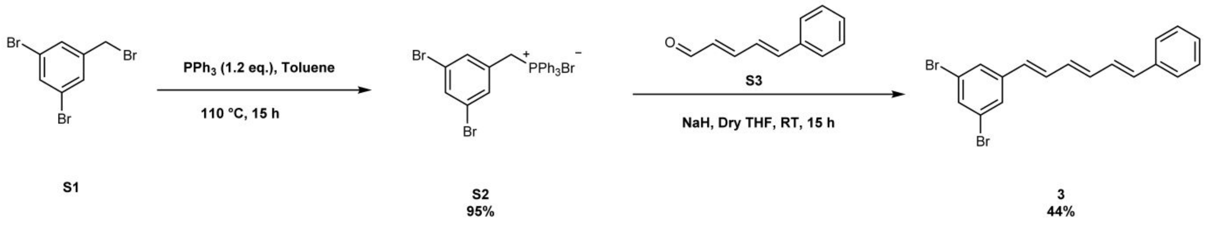

(3,5-dibromobenzyl)triphenylphosphonium bromide [S2]

Adapted from literatures procedures.[1,9]

To a flask set up for reflux was added 3,5-dibromobenzylbromide, **S1,** (2.0 g, 6.1 mmol, 1 equiv.), triphenylphosphine (1.9 g, 7.3 mmol, 1.2 equiv.) and toluene (~30 mL). The mixture was refluxed at 110 ^{o}C for 15 h. The reaction was then cooled to RT, to 0 °C and the resulting precipitate was filtered under vacuum. The filter cake was washed with toluene (× 2) and hexanes (× 2) and then dried under vacuum to afford the title product, **S2**, as a white powder, (3.4 g, 5.8 mmol, 95%). ^{1}H NMR data is consistent with the literature.[10]

^{1}H NMR (400 MHz $CDCl_3$) δ 7.87 – 7.78 (m, 9H), 7.67 – 7.63 (m, 6H), 7.47 (m, 1H), 7.16 (m, 2H), 5.72 (d, J = 14.9 Hz, 2H).

1,3-dibromo-5-((1E,3E,5E)-6-phenylhexa-1,3,5-trien-1-yl)benzene [3]

3 was synthesized by a Wittig reaction of pentadienal **S3** (prepared as previously reported)[11] and the phosphonium salt, **S2**. Sodium hydride (60% dispersion in mineral oil, 96 mg, 2.4 mmol, 1.85 eq.) was dissolved in dry THF (25 mL) and cooled to 0 °C using an ice bath, under argon. Under a positive flow of argon, **S2** (1.0 g, 1.7 mmol, 1.25 eq.) was added as a single solid portion. The ice bath was removed, and the suspension was stirred at room temperature for 15 minutes. A solution of **S3** (214 mg, 1.3 mmol, 1.0 eq.) in dry THF (5 mL) was prepared and added dropwise and the mixture stirred overnight, in the dark. The mixture was poured over ice/brine, diluted with EtOAc and the layers separated. The organic layer was washed with 1 M HCl (10 mL) and brine (10 mL), dried ($MgSO_4$) and the solvent removed *in vacuo.* The solid material was sonicated in methanol and filtered. **3** was obtained as a pale-yellow powder (226 mg, 0.58 mmol, 44%).

^{1}H NMR (500 MHz, CD_2Cl_2) δ 7.51 (m, 3H), 7.44 (d, J = 7.3 Hz, 2H), 7.34 (t, J = 7.3 Hz, 2H), 7.4 (d, J = 7.3 Hz, 1H), 6.91 (m, 2H), 6.70 – 6.42 (m, 4H).

^{13}C NMR (126 MHz, CD_2Cl_2) δ 141.3, 137.1, 135.7, 134.0, 132.5, 132.3, 132.0, 129.1, 128.7, 127.9, 126.5, 123.1.

HRMS(m/z) Found [M+H]+ = 388.9534, $C_{18}H_{15}Br_2$ requires 388.9541, Δ = -0.7 ppm

Scheme S2. Synthesis of a contiguous DPH dimer and both branched and linear trimers. R = 2-ethylhexyl.

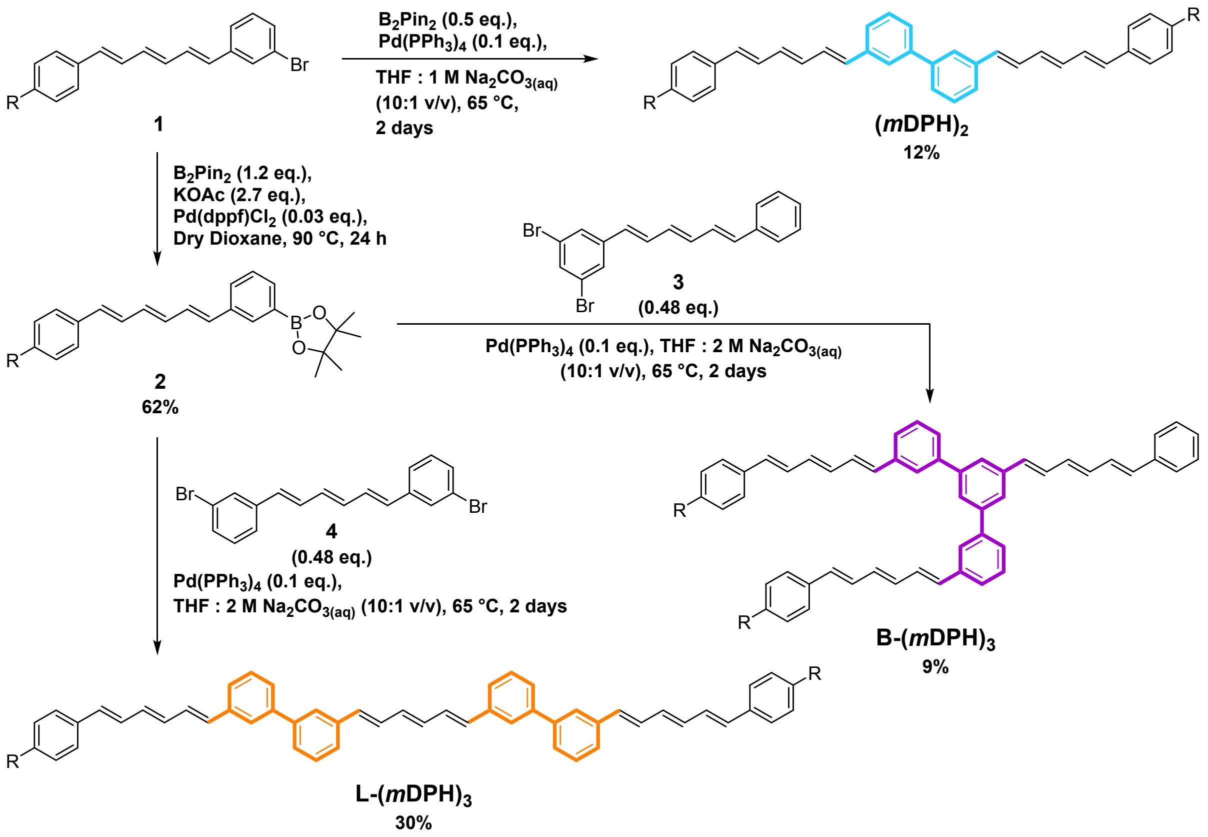

3,3'-bis((1E,3E,5E)-6-(4-(2-ethylhexyl)phenyl)hexa-1,3,5-trien-1-yl)-1,1'-biphenyl [(*m*DPH)$_2$]

A microwave vial was charged with a stir bar, **1** (100 mg, 0.24 mmol, 1.0 eq.) (synthesized as previously reported)[1], bis(pinacolato)diboron (30 mg, 0.12 mmol, 0.5 eq.), and $Pd(PPh_3)_4$ (27 mg, 0.024 mmol, 0.1 eq.) and flushed under argon. THF (7 mL) and an aqueous solution of Na_2CO_3 (1M, 0.7 mL) were separately degassed and then added to the solid reagents. The reaction mixture was lowered into a preheated oil bath and heated at 65 °C in the dark for 2 days. The reaction mixture was carefully acidified with 1 M HCl (~ 3 mL) with stirring and then diluted with DCM (~75 mL) and brine (~50 mL). The organic layer was separated and washed with brine (~ 20 mL), dried ($MgSO_4$) and the solvent remove *in vacuo*. The crude material was then purified by flash column chromatography (eluent: DCM/ *n*-hexane gradient from 0:100 → 10:0 v/v). Following removal of the solvent the columned material was sonicated in hexane to obtain the product, **(*m*DPH)$_2$**, as an off-white powder (20 mg, 0.029 mmol, 12%).

^{1}H NMR (500 MHz, $CDCl_3$) δ 7.65 (s, 2H), 7.48 – 7.42 (m, 6H), 7.36 (d, *J* = 8.2 Hz, 4H), 7.13 (d, *J* = 8.2 Hz, 4H), 7.02 – 6.97 (m, 2H), 6.92 – 6.87 (m, 2H), 6.68 (d, *J* = 15.5 Hz, 2H), 6.62 (d, *J* = 15.4 Hz, 2H), 6.59 – 6.52 (m, 4H), 2.56 – 2.48 (m, 4H), 1.58 – 1.53 (m, 2H), 1.33 – 1.24 (m, 16H), 0.90 – 0.86 (m, 12H).

13C NMR (176 MHz, CD_2Cl_2) δ 142.3, 141.8, 138.4, 135.1, 134.6, 133.4, 133.3, 132.5, 130.1, 130.0, 129.5, 128.5, 126.7, 126.5, 125.7, 125.5, 41.5, 40.2, 32.7, 29.2, 25.8, 23.5, 14.3, 11.0.

HRMS(m/z) Found [M+H]+ = 687.4915, $C_{52}H_{62}$ requires 687.4930, Δ = -1.5 ppm

2-(3-((1E,3E,5E)-6-(4-(2-ethylhexyl)phenyl)hexa-1,3,5-trien-1-yl)phenyl)-4,4,5,5-tetramethyl-1,3,2-dioxaborolane [2]

2 was prepared by a Miyaura borylation from the bromide **1**. **1** (846 mg, 2.0 mmol, 1.0 eq.), bis(pinacolato)diboron (609 mg, 2.4 mmol, 1.2 eq.), Pd(dppf)Cl_2 (44 mg, 0.06 mmol, 0.03 eq.), potassium acetate (530 mg, 5.4 mmol, 2.7 eq.) and stir bar were added to a Schlenk flask, which was evacuated and backfilled with argon 5 times. Dry dioxane (40 mL) was added, the reaction mixture was lowered into a preheated oil bath and stirred at 90 °C in the dark for 24 h. The reaction mixture was allowed to cool to room temperature and then the solvent removed *in vacuo*. The crude material was dissolved in DCM, dry loaded onto silica and purified by flash column chromatography (eluent: DCM/ *n*-hexane gradient from 0:100 → 30:70 v/v). After removal of the solvent, the material was sonicated in MeOH, filtered and dried under suction to obtain the product, **2**, as an off-white powder (580 mg, 1.23 mmol, 62%).

^{1}H NMR (500 MHz, $CDCl_3$) δ 7.86 (s, 1H), 7.66 (d, J = 7.3 Hz, 1H), 7.50 (d, J = 7.8 Hz, 1H), 7.34 – 7.32 (m, 3H), 7.10 (d, J = 7.7 Hz, 2H), 6.96 – 6.91 (m, 1H), 6.87 – 6.82 (m, 1H), 6.61 – 6.46 (m, 4H), 2.58 – 2.50 (m, 2H), 1.61 – 1.54 (m, 1H), 1.36 (s, 12H), 1.34 – 1.26 (m, 8H), 0.91 – 0.88 (m, 6H).

^{13}C NMR (126 MHz, $CDCl_3$): δ 141.8, 137.0, 134.9, 134.0, 133.9, 133.3, 132.9, 132.9, 132.4, 129.7, 129.5, 129.3, 128.4, 128.2, 126.3, 84.0, 41.2, 40.1, 32.5, 29.0, 25.6, 25.0, 23.2, 14.3, 10.9.

HRMS(m/z) Found [M+H]+ = 471.3429, $C_{32}H_{43}BO_2$ requires 471.3434, Δ = -0.5 ppm

3,3''-bis((1E,3E,5E)-6-(4-(2-ethylhexyl)phenyl)hexa-1,3,5-trien-1-yl)-5'-((1E,3E,5E)-6-phenylhexa-1,3,5-trien-1-yl)-1,1':3',1''-terphenyl [B-(*m*DPH)$_3$]

A 10 mL microwave vial was charged with a stir bar, **2** (282 mg, 0.60 mmol, 2.1 eq.), **3** (111 mg, 0.29 mmol, 1.0 eq.), and Pd(PPh_3)$_4$ (35 mg, 0.03 mmol, 0.1 eq.) and flushed under argon. THF (4 mL) and an aqueous solution of Na_2CO_3 (2M, 0.9 mL) were separately degassed and then added to the solid

reagents. The reaction mixture was lowered into a preheated oil bath and heated at 65 °C in the dark for 2 days. The reaction mixture was carefully acidified with 1 M HCl (~ 4 mL) with stirring and then diluted with DCM (~100 mL) and brine (~75 mL). The organic layer was separated and washed with brine (~ 30 mL), dried ($MgSO_4$) and the solvent remove *in vacuo*. The crude material was then purified by flash column chromatography (eluent: DCM/ *n*-hexane gradient from 0:100 → 35:70 v/v). Following removal of the solvent from the fractions containing only the product (identified by TLC) the material was sonicated in MeOH and the solid filtered to obtain **B-(*m*DPH)$_3$** as a pale yellow powder (25 mg, 0.027 mmol, 9%).

1H NMR (700 MHz, CD_2Cl_2) δ 7.75 (s, 2H), 7.74 (s, 1H), 7.68 (d, J = 1.7 Hz, 2H), 7.56 (dt, J = 6.9, 1.7 Hz, 2H), 7.48 – 7.44 (m, 6H), 7.35 – 7.33 (m, 6H), 7.24 (t, J = 7.3 Hz, 1H), 7.12 (d, J = 8.0 Hz, 4H), 7.11 – 7.09 (m, 1H), 7.06 – 7.02 (m, 2H), 6.98 – 6.94 (m, 1H), 6.92 – 6.88 (m, 2H), 6.78 (d, J = 15.5 Hz, 1H), 6.71 (d, J = 15.5 Hz, 2H), 6.66 (d, J = 15.5 Hz, 1H), 6.63 – 6.55 (m, 8H), 2.55 – 2.50 (m, 4H), 1.60 – 1.54 (m, 2H), 1.33 – 1.20 (m, 16H), 0.89 – 0.87 (m, 12H).

13C NMR (176 MHz, CD_2Cl_2) δ 142.4, 142.3, 141.8, 138.9, 138.5, 137.8, 135.1, 134.6, 134.5, 133.9, 133.4, 133.4, 133.3, 132.7, 132.4, 130.4, 130.2, 130.0, 129.6, 129.5, 129.1, 128.5, 128.0, 126.8, 126.5, 126.0, 125.8, 125.6, 124.6, 41.5, 40.2, 32.7, 29.2, 25.8, 23.5, 14.3, 11.0.

HRMS(m/z) Found [M+H]+ = 917.6021, $C_{70}H_{76}$ requires 917.6025, Δ = -0.4 ppm

(1E,3E,5E)-1,6-bis(3'-((1E,3E,5E)-6-(4-(2-ethylhexyl)phenyl)hexa-1,3,5-trien-1-yl)-[1,1'-biphenyl]-3-yl)hexa-1,3,5-triene [L-(*m*DPH)$_3$]

L-(*m*DPH)$_3$ was prepared by an analogous Suzuki coupling procedure to the preparation of **B-(*m*DPH)$_3$** on the same scale using: **2** (282 mg, 0.60 mmol, 2.1 eq.), **4** (111 mg, 0.29 mmol, 1.0 eq.) (prepared as previously reported),[11] $Pd(PPh_3)_4$ (35 mg, 0.03 mmol, 0.1 eq.), THF (4 mL), and an aqueous solution of Na_2CO_3 (2M, 0.9 mL). Following the work-up and purification steps **L-(*m*DPH)$_3$** was obtained as a yellow powder (83 mg, 0.091 mmol, 31%).

^{1}H NMR (500 MHz, $CDCl_3$) δ 7.64 (m, 4H), 7.47 – 7.40 (m, 12H), 7.34 (d, *J* = 8.2 Hz, 4H), 7.11 (d, *J* = 8.2 Hz, 4H), 7.02 – 6.95 (m, 4H), 6.89 – 6.85 (m, 2H), 6.71 – 6.50 (m, 12H), 2.55 – 2.49 (m, 4H), 1.58 (m, 2H), 1.35 – 1.23 (m, 16H), 0.91 – 0.86 (m, 12H).

^{13}C NMR (126 MHz, $CDCl_3$): δ 141.7, 141.6, 138.0, 137.9, 134.7, 134.2, 133.8, 133.0, 132.9, 132.7, 132.1, 129.7, 129.5, 129.1, 128.2, 126.5, 126.4, 126.2, 125.4, 125.3, 125.3, 125.2, 41.2, 40.1, 32.5, 29.0, 25.6, 23.2, 14.3, 10.9.

HRMS(m/z) Found [M+H]+ = 917.5987, $C_{70}H_{76}$ requires 917.6025, Δ = -3.8 ppm

4. NMR Spectra

(3,5-dibromobenzyl)triphenylphosphonium bromide [S2]

^{1}H NMR, 400 MHz, $CDCl_3$

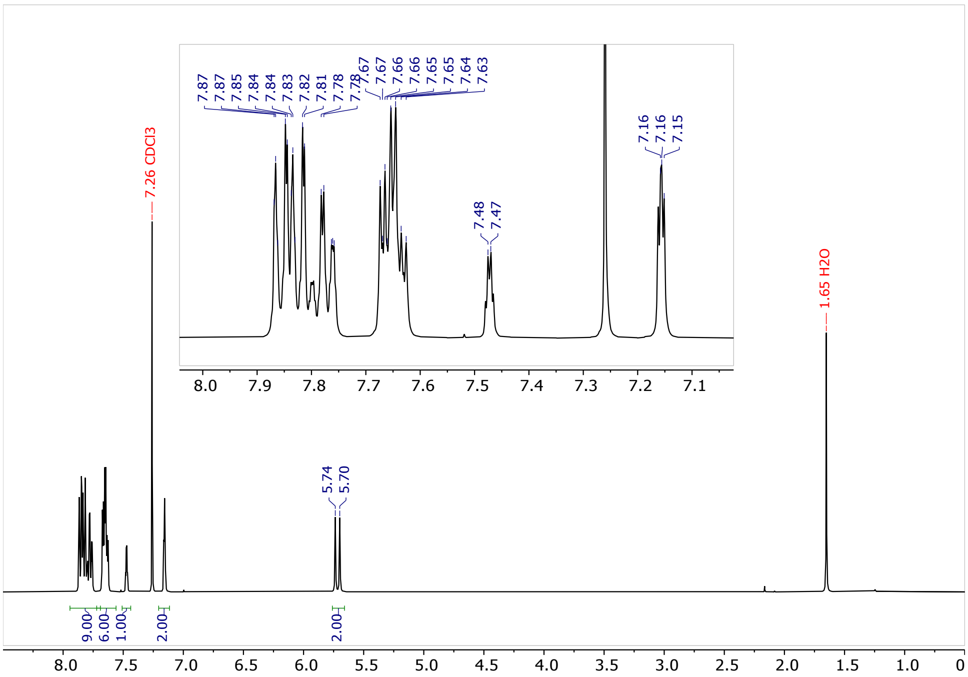

1,3-dibromo-5-((1E,3E,5E)-6-phenylhexa-1,3,5-trien-1-yl)benzene [3]

^{1}H NMR, 500 MHz, CD_2Cl_2

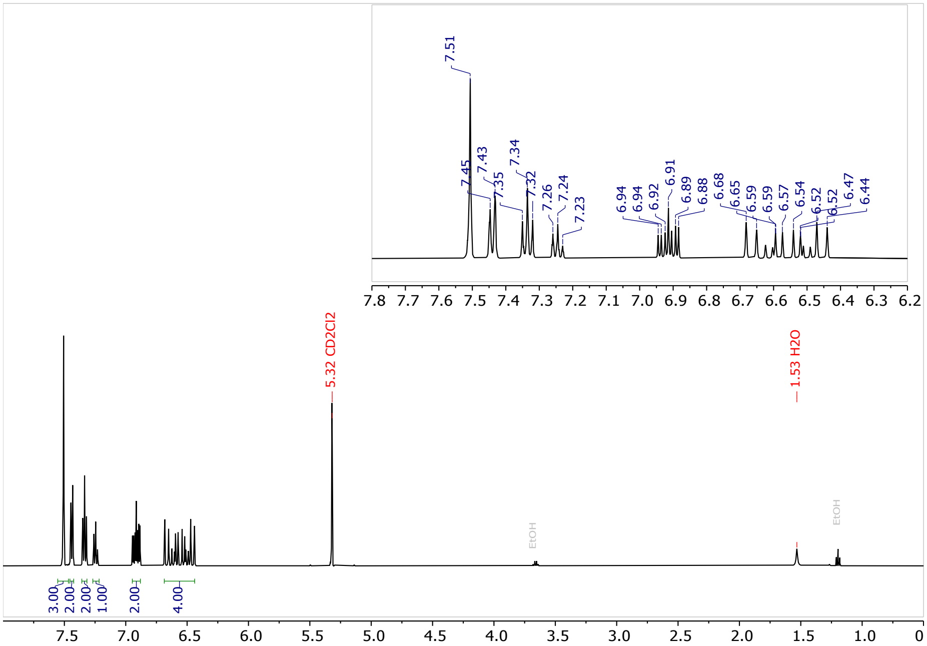

^{13}C NMR, 126 MHz, CD_2Cl_2

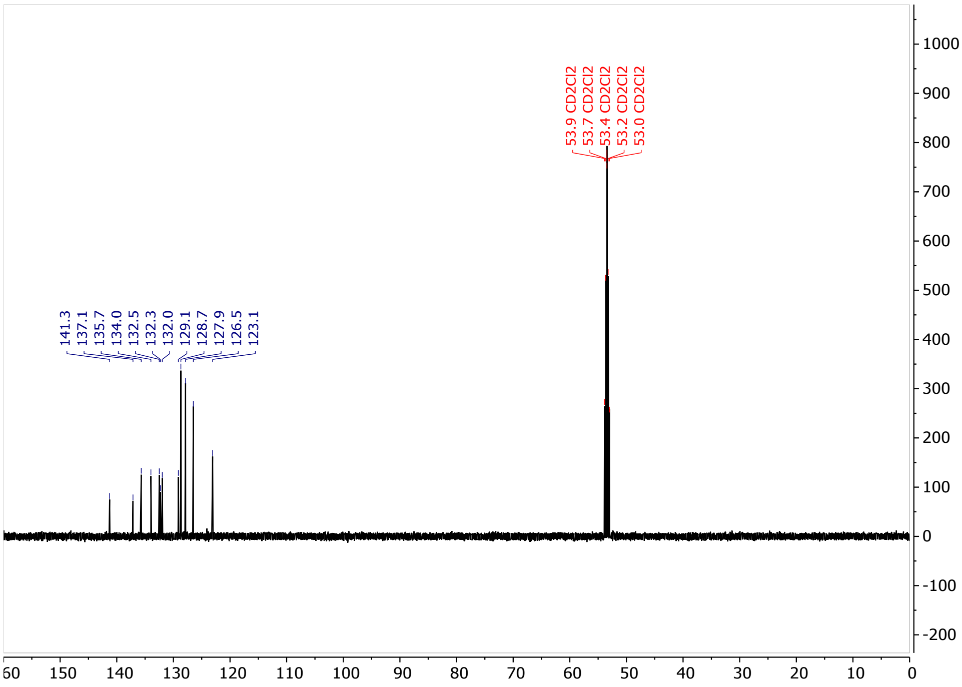

3,3'-bis((1E,3E,5E)-6-(4-(2-ethylhexyl)phenyl)hexa-1,3,5-trien-1-yl)-1,1'-biphenyl [(*m*DPH)$_2$]

^{1}H NMR, 500 MHz, $CDCl_3$

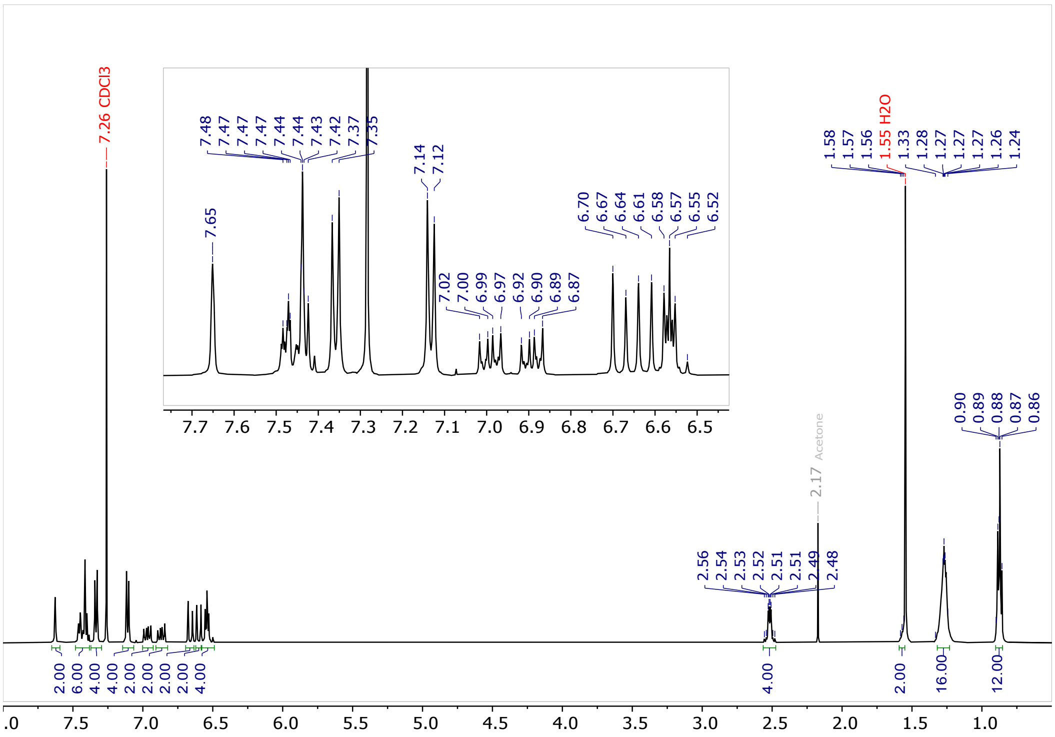

^{13}C NMR, 176 MHz, CD_2Cl_2

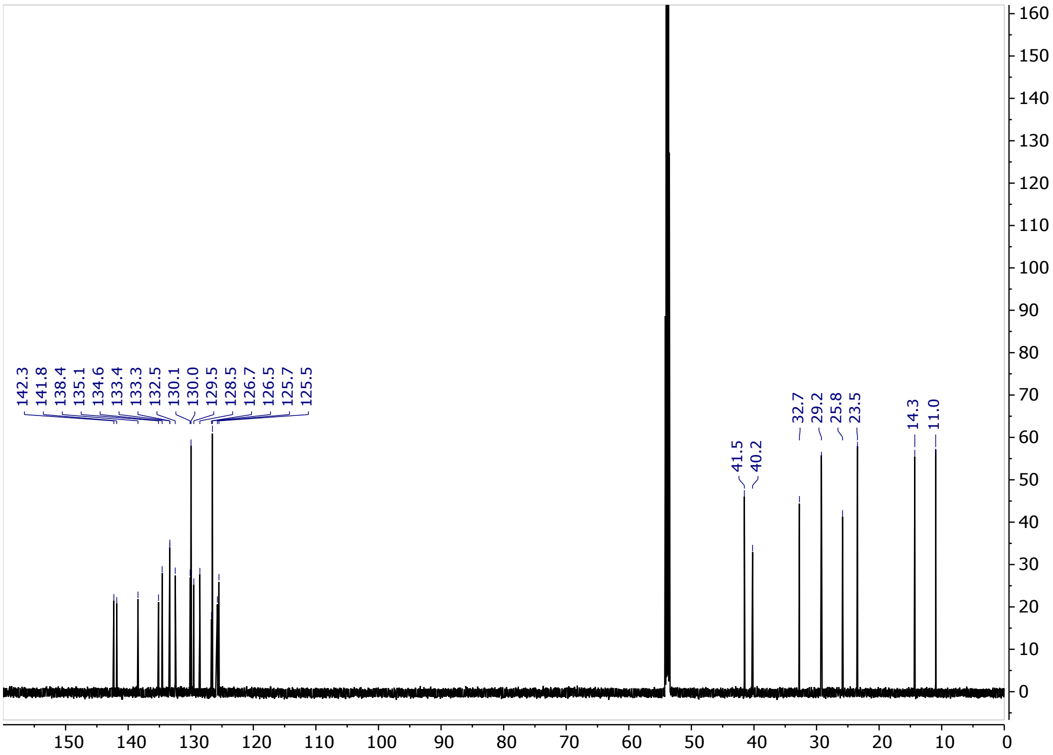

2-(3-((1E,3E,5E)-6-(4-(2-ethylhexyl)phenyl)hexa-1,3,5-trien-1-yl)phenyl)-4,4,5,5-tetramethyl-1,3,2-dioxaborolane [2]

^{1}H NMR, 500 MHz, $CDCl_3$

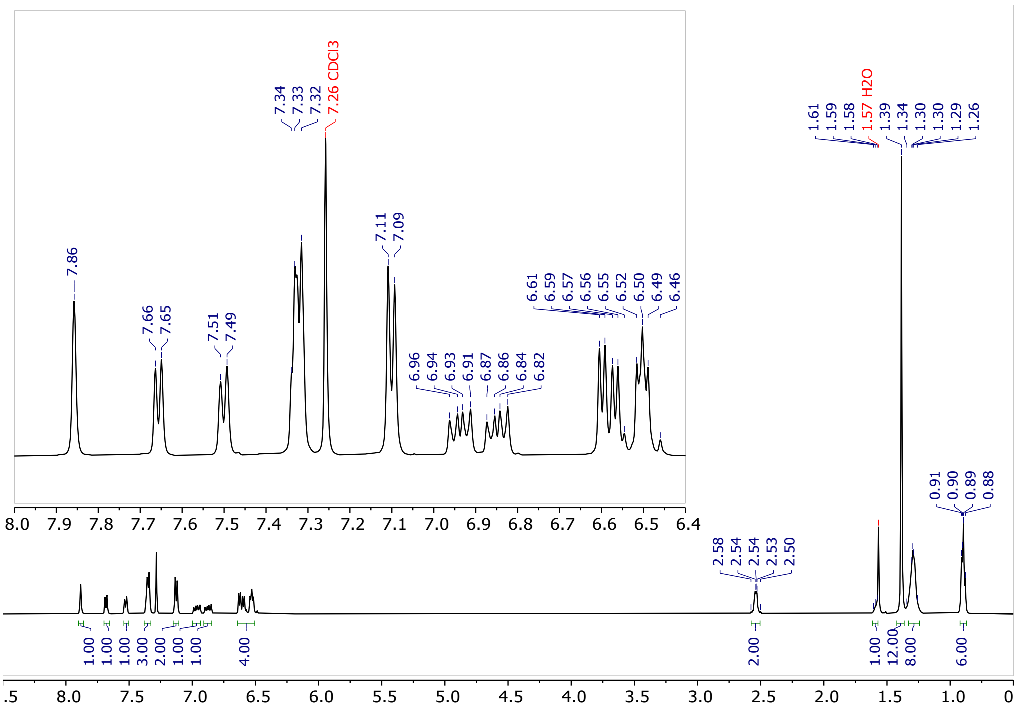

^{13}C NMR, 126 MHz, $CDCl_3$

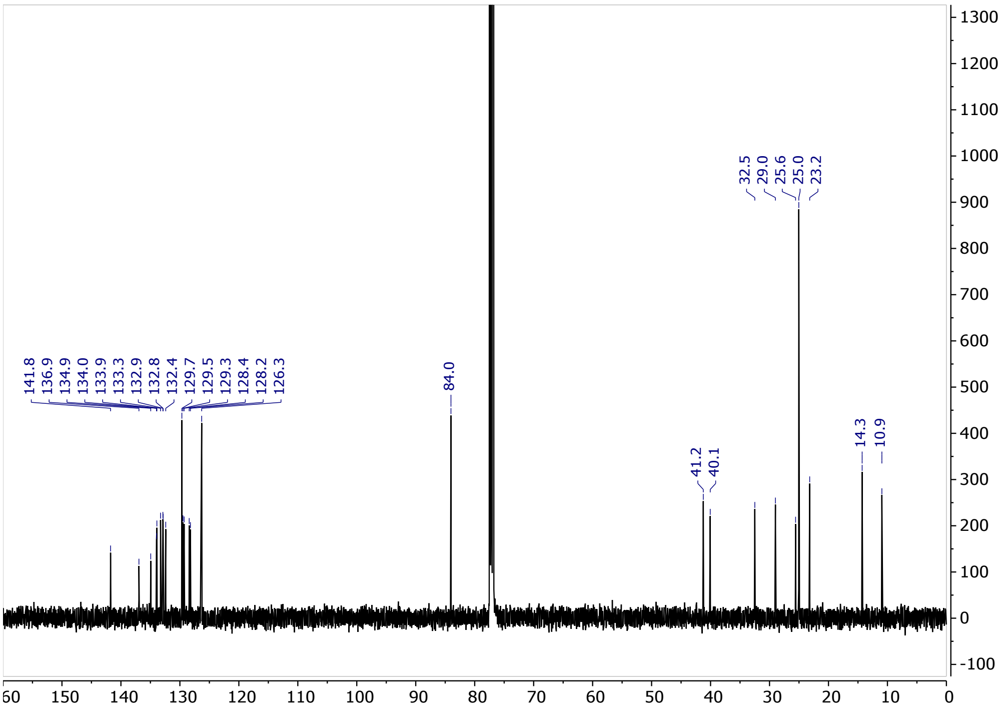

3,3''-bis((1E,3E,5E)-6-(4-(2-ethylhexyl)phenyl)hexa-1,3,5-trien-1-yl)-5'-((1E,3E,5E)-6-phenylhexa-1,3,5-trien-1-yl)-1,1':3',1''-terphenyl [B-(*m*DPH)$_3$]

^{1}H NMR, 700 MHz, CD_2Cl_2

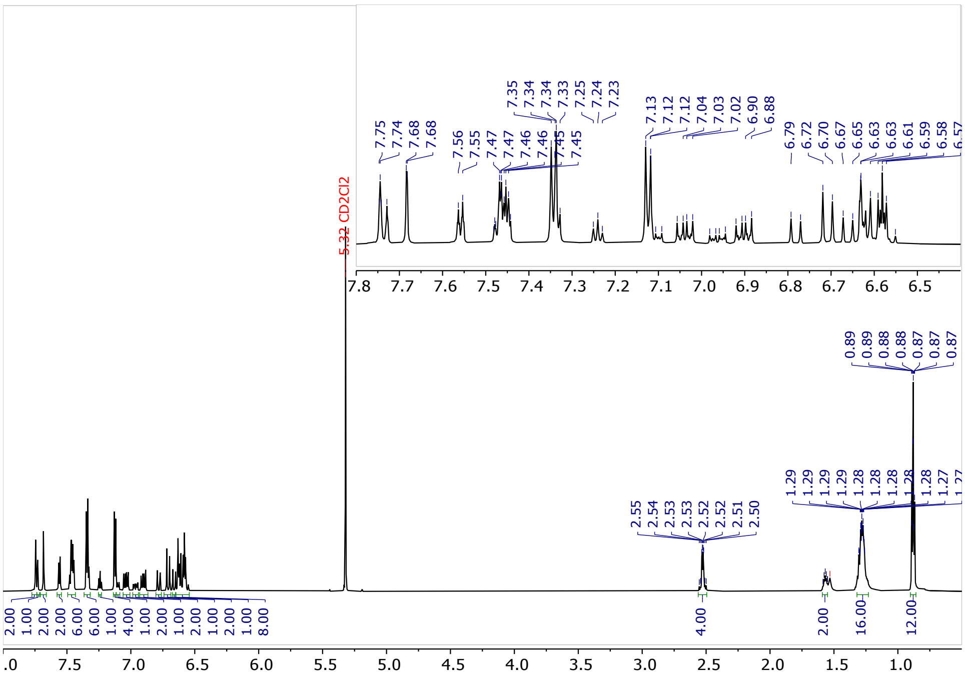

^{13}C NMR, 176 MHz, CD_2Cl_2

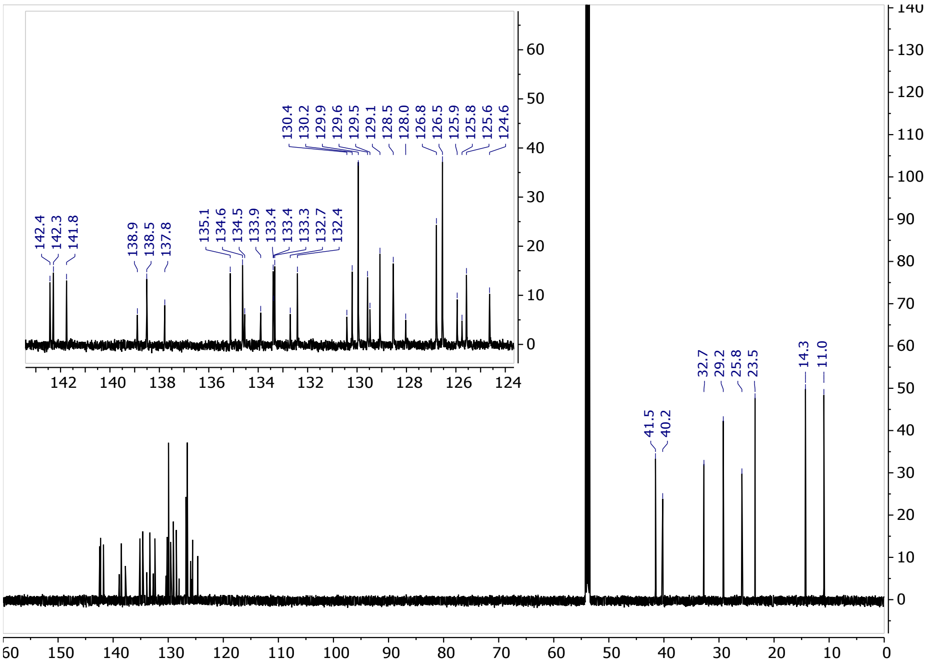

(1E,3E,5E)-1,6-bis(3'-((1E,3E,5E)-6-(4-(2-ethylhexyl)phenyl)hexa-1,3,5-trien-1-yl)-[1,1'-biphenyl]-3-yl)hexa-1,3,5-triene [L-(*m*DPH)$_3$]

^{1}H NMR, 500 MHz, $CDCl_3$

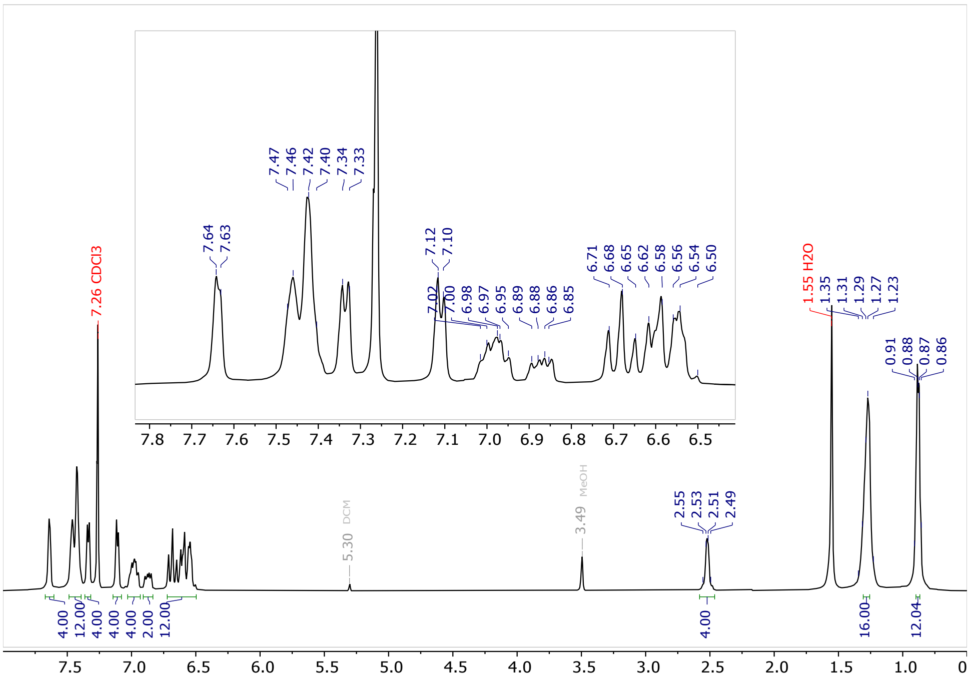

^{13}C NMR, 126 MHz, $CDCl_3$

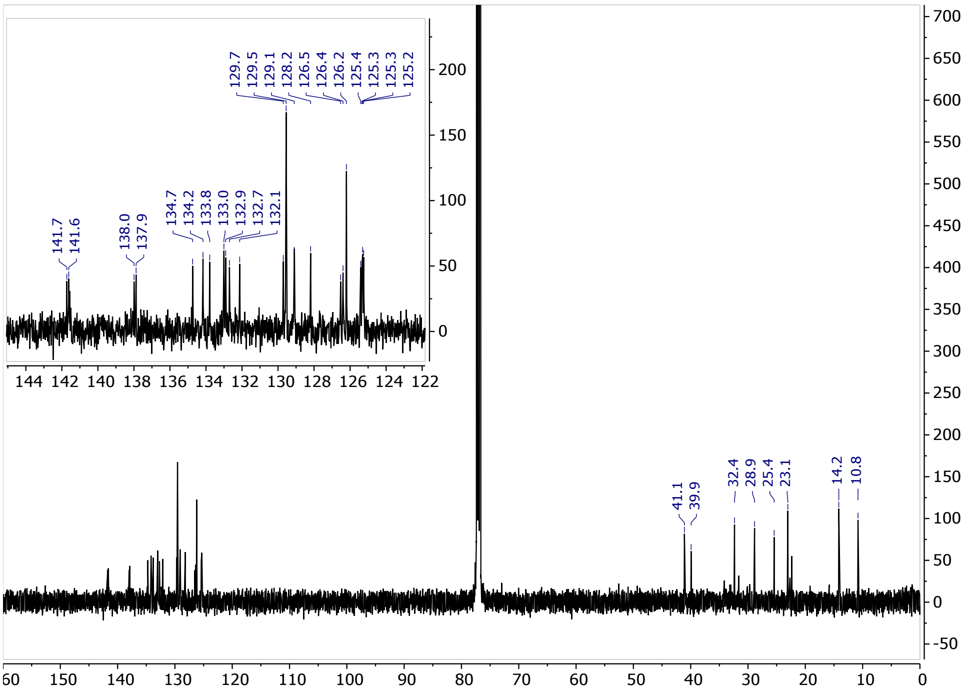

5. Mass Spectra of Oligomers

3,3'-bis((1E,3E,5E)-6-(4-(2-ethylhexyl)phenyl)hexa-1,3,5-trien-1-yl)-1,1'-biphenyl [(*m*DPH)$_2$]

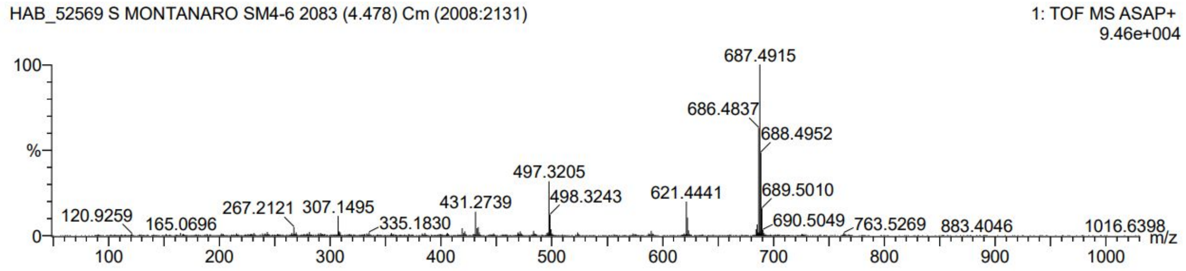

Minimum:				-1.5				
Maximum:		5.0	500.0	50.0				
Mass	Calc. Mass	mDa	PPM	DBE	i-FIT	Norm	Conf(%)	Formula
687.4915	687.4930	-1.5	-2.2	21.5	356.7	n/a	n/a	C52 H63

3,3''-bis((1E,3E,5E)-6-(4-(2-ethylhexyl)phenyl)hexa-1,3,5-trien-1-yl)-5'-((1E,3E,5E)-6-phenylhexa-1,3,5-trien-1-yl)-1,1':3',1''-terphenyl [B-(*m*DPH)$_3$]

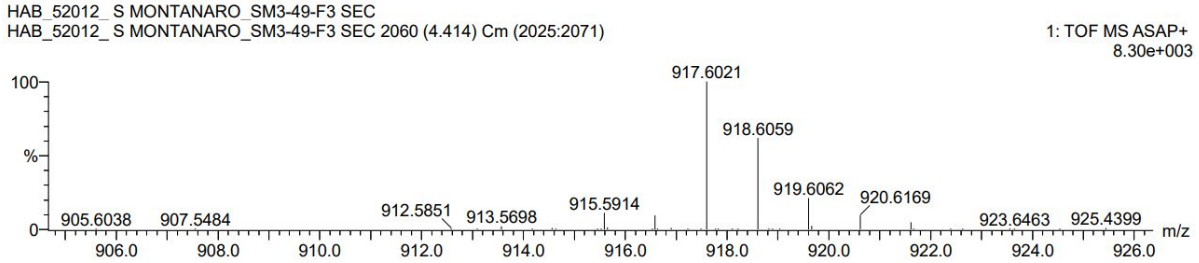

Minimum:				-1.5				
Maximum:		5.0	3000.0	50.0				
Mass	Calc. Mass	mDa	PPM	DBE	i-FIT	Norm	Conf(%)	Formula
917.6021	917.6025	-0.4	-0.4	32.5	76.5	n/a	n/a	C70 H77

(1E,3E,5E)-1,6-bis(3'-((1E,3E,5E)-6-(4-(2-ethylhexyl)phenyl)hexa-1,3,5-trien-1-yl)-[1,1'-biphenyl]-3-yl)hexa-1,3,5-triene [L-(*m*DPH)$_3$]

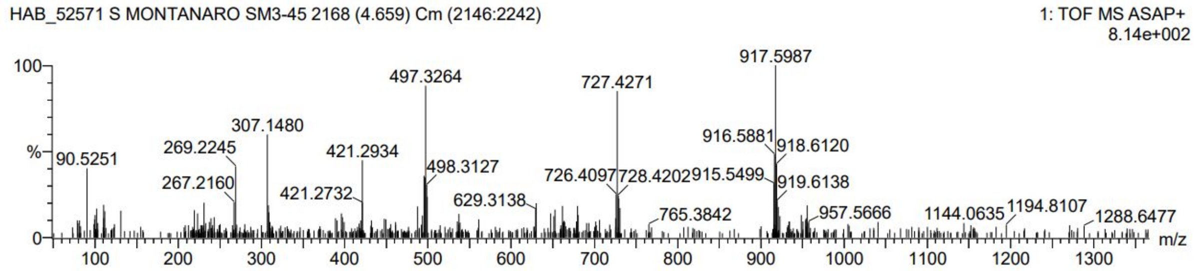

Minimum:				-1.5				
Maximum:		5.0	500.0	50.0				
Mass	Calc. Mass	mDa	PPM	DBE	i-FIT	Norm	Conf(%)	Formula
917.5987	917.6025	-3.8	-4.1	32.5	59.1	n/a	n/a	C70 H77

6. References

(1) Millington, O.; Montanaro, S.; Leventis, A.; Sharma, A.; Dowland, S. A.; Sawhney, N.; Fallon, K. J.; Zeng, W.; Congrave, D. G.; Musser, A. J.; Rao, A.; Bronstein, H. Soluble Diphenylhexatriene Dimers for Intramolecular Singlet Fission with High Triplet Energy. *J Am Chem Soc* **2023**, *145* (4), 2499–2510. https://doi.org/10.1021/jacs.2c12060.

(2) Chattopadhyay, S. K.; Das, P. K.; Hug, G. L. Photoprocesses in Diphenylpolyenes. Oxygen and Heavy-Atom Enhancement of Triplet Yields. *J Am Chem Soc* **1982**, *104* (17), 4507–4514. https://doi.org/10.1021/ja00381a001.

(3) Wang, Z.; Liu, H.; Xie, X.; Zhang, C.; Wang, R.; Chen, L.; Xu, Y.; Ma, H.; Fang, W.; Yao, Y.; Sang, H.; Wang, X.; Li, X.; Xiao, M. Free-Triplet Generation with Improved Efficiency in Tetracene Oligomers through Spatially Separated Triplet Pair States. *Nat Chem* **2021**, *13* (6), 559–567. https://doi.org/10.1038/s41557-021-00665-7.

(4) Liu, H.; Wang, Z.; Wang, X.; Shen, L.; Zhang, C.; Xiao, M.; Li, X. Singlet Exciton Fission in a Linear Tetracene Tetramer. *J Mater Chem C Mater* **2018**, *6* (13), 3245–3253. https://doi.org/10.1039/C7TC05783K.

(5) Liu, H.; Wang, R.; Shen, L.; Xu, Y.; Xiao, M.; Zhang, C.; Li, X. A Covalently Linked Tetracene Trimer: Synthesis and Singlet Exciton Fission Property. *Org Lett* **2017**, *19* (3), 580–583. https://doi.org/10.1021/acs.orglett.6b03739.

(6) Korovina, N. V.; Chang, C. H.; Johnson, J. C. Spatial Separation of Triplet Excitons Drives Endothermic Singlet Fission. *Nat Chem* **2020**, *12* (4), 391–398. https://doi.org/10.1038/s41557-020-0422-7.

(7) Nakamura, S.; Sakai, H.; Fuki, M.; Ooie, R.; Ishiwari, F.; Saeki, A.; Tkachenko, N. V.; Kobori, Y.; Hasobe, T. Thermodynamic Control of Intramolecular Singlet Fission and Exciton Transport in Linear Tetracene Oligomers. *Angewandte Chemie* **2022**. https://doi.org/10.1002/ange.202217704.

(8) Nakamura, S.; Sakai, H.; Nagashima, H.; Fuki, M.; Onishi, K.; Khan, R.; Kobori, Y.; Tkachenko, N. V.; Hasobe, T. Synergetic Role of Conformational Flexibility and Electronic Coupling for Quantitative Intramolecular Singlet Fission. *The Journal of Physical Chemistry C* **2021**, *125* (33), 18287–18296. https://doi.org/10.1021/acs.jpcc.1c04734.

(9) Ramachandran, G. K.; Tomfohr, J. K.; Li, J.; Sankey, O. F.; Zarate, X.; Primak, A.; Terazono, Y.; Moore, T. A.; Moore, A. L.; Gust, D.; Nagahara, L. A.; Lindsay, S. M. Electron Transport Properties of a Carotene Molecule in a Metal-(Single Molecule)-Metal Junction. *Journal of Physical Chemistry B* **2003**, *107* (25), 6162–6169. https://doi.org/10.1021/jp0343786.

(10) Plater, M. J. Fullerene Tectonics. Part 2.1 Synthesis and Pyrolysis of Halogenated Benzo[c]Phenanthrenes. *J Chem Soc Perkin 1* **1997**, No. 19, 2903–2909. https://doi.org/10.1039/a701916e.

(11) Millington, O.; Sharma, A.; Montanaro, S.; Leventis, A.; Dowland, S. A.; Congrave, D. G.; Lee, C.-A.; Rao, A.; Bronstein, H. Synthesis and Intramolecular Singlet Fission Properties of *Ortho* -Phenylene Linked Oligomers of Diphenylhexatriene. *Chem Sci* **2023**, *14* (45), 13090–13094. https://doi.org/10.1039/D3SC03665K.
